# A synopsis of neotropical parasitoid wasps of *Diaphania hyalinata* (Lepidoptera, Crambidae), with new records from Guadeloupe

**DOI:** 10.3897/zookeys.1286.189299

**Published:** 2026-07-29

**Authors:** Margot Gumbau, Claire Marteil, Géraldine Groussier, Astrid Cruaud, Sabine Nidelet, Sylvie Warot, José Fernandez-Triana, Laurent Penet, Jean-Marc Blazy, Jean-Yves Rasplus

**Affiliations:** 1 INRAE, UR ASTRO, Petit-Bourg, Guadeloupe, France CBGP, INRAE, CIRAD, IRD, Montpellier SupAgro, Univ Montpellier Montpellier France; 2 UMR ISA, INRAE, Université Côte d’Azur, Sophia-Antipolis, France INRAE, UR ASTRO Petit-Bourg Guadelupe (Fr); 3 CBGP, INRAE, CIRAD, IRD, Montpellier SupAgro, Univ Montpellier, Montpellier, France UMR ISA, INRAE, Université Côte d’Azur Sophia-Antipolis France; 4 Canadian National Collection of Insects, Ottawa, Canada Canadian National Collection of Insects Ottawa Canada

**Keywords:** Barcoding, biocontrol, Chalcidoidea, entomology, Ichneumonoidea, melonworm, tropical entomology

## Abstract

*Diaphania hyalinata* Linnaeus, 1767 (Lepidoptera, Crambidae), commonly known as the melonworm, is a major pest of cucurbits in the Neotropics. Yet, a comprehensive review of the parasitoid wasps that may act as biocontrol agents of this pest is still lacking. An extensive literature review was conducted to compile the current knowledge on the identity, distribution, and biology of parasitoid wasps associated with *D. hyalinata* across its range. Eggs, larvae, and pupae of *D. hyalinata* were collected in Guadeloupe and reared to document the local parasitoid diversity. Among the 37 parasitoids identified in our review, eight were recovered during our field survey, all of which are new records for the island. These parasitoids were photographed and DNA-barcoded to facilitate their potential use in future biological control programs targeting *D. hyalinata*. The species *Trichogramma pretiosa* Riley, 1879 (Trichogrammatidae; egg parasitoid) and *Schoenlandella montserratensis* Kang, 2021 (Braconidae; larval parasitoid) appear to be promising candidates for the biological control of *D. hyalinata* in Guadeloupe. It may be also worth evaluating the effectiveness of two additional larval parasitoids, *Eiphosoma dentator* Fabricius, 1804 (Ichneumonidae) and *Apanteles impiger* Muesebeck, 1958 (Braconidae), while assessing possible non-target effects on local fauna. However, it is important to consider that the presence of the hyperparasitoid *Aphanogmus fijiensis* Ferrière, 1933 (Ceraphronidae) could undermine biocontrol efforts. Overall, it would be valuable to gain a better understanding of the biological and environmental factors (such as agricultural practices and landscape features) that enhance the natural regulatory activities of this parasitoid community.

## Introduction

*Diaphania hyalinata*, Linnaeus, 1767 (Lepidoptera, Crambidae), commonly known as the melonworm, is a major pest of cucurbits. The species is widely distributed in the Neotropics (Panthi et al. 2017; Capote Luna et al. 2019) and also reaches the Nearctic, ranging from Ontario (Canada) in the north to Buenos Aires province (Argentina) in the south, including the Caribbean (Acosta et al. 1986; Guillaume and Boissot 2001).

The larvae of *D. hyalinata* primarily feed on leaves, causing significant defoliation. The resulting reduction in leaf area decreases the plant’s photosynthetic capacity, ultimately leading to yield losses (Gonring et al. 2003). As populations increase, larvae become less selective and may feed on all aerial parts of the plant (Valles and Capinera 1992). In Florida, yield losses of up to 23% have been reported on yellow squash due to damage caused by *D. hyalinata* (McSorley and Waddill 1982). A related species, *Diaphania indica* (Saunders, 1851), found in Africa and Asia, has also been associated with significant yield reductions when more than one larva per leaf is present (Schreiner 1991). These observations confirm the status of *D. hyalinata* as a major cucurbit pest, with substantial impact on crop yields.

In Guadeloupe, a French island in the French West Indies, cucumber (*Cucumis sativus* L., 1753) accounts for nearly 10% of both the total area (ha) and production (metric tons) dedicated to market gardening (DAAF Guadeloupe 2023), and *D. hyalinata* is considered its most damaging pest. The life cycle of *D. hyalinata* spans ~30 days, with the larval stage (from L1 to pupation) lasting ~14 days (Capinera 2003). Warmer temperatures shorten the life cycle, leading to increased pest pressure (Ba‐Angood 1979; Melo et al. 2007). The presence of *D. hyalinata* is also significantly correlated with humidity (Barceló 2003; Jn and Venkatesha 2022). In tropical environments such as Guadeloupe, climatic conditions create an ideal context for severe infestations all year, emphasizing the need for effective and locally adapted control strategies.

Parasitoid wasps are considered key biological control agents, as females lay their eggs in or on pest hosts, and the host eventually dies when the adult wasp emerges (Godfray 1994). These wasps include a wide variety of families, genera, and species that naturally contribute to pest regulation.

Parasitoid regulation of *D. hyalinata* has been observed across its life stages and throughout its distribution, but the associated species appear to vary regionally. To develop agroecological control strategies that support the action of local parasitoids, it is essential to first identify which species are present and understand their biology. Yet, a comprehensive review of these parasitoid complexes across the neotropics is still lacking.

In this study, we conducted an extensive literature review to compile current knowledge on the identity, distribution, and biology of parasitoids associated with *D. hyalinata* across its range. We then collected eggs, larvae, and pupae of *D. hyalinata* in Guadeloupe and reared them to document the local parasitoid diversity. Recovered parasitoids were photographed and DNA-barcoded to facilitate future implementation of biological control programs targeting *D. hyalinata* on the island.

## Materials and methods

### Literature-based synopsis of *D. hyalinata* parasitoids

The synopsis of *D. hyalinata* parasitoids was compiled using a digitized and OCR-processed corpus of literature, including reprints, books, peer-reviewed articles, extension documents, and other sources, amounting to more than 55,000 references. This corpus represents a compilation of the most significant literature on hymenopteran parasitoids. The initial search was performed using the key words *‘Diaphania hyalinata* Neotropical parasitoids’. Once the parasitoid species were identified, both their valid names and known synonyms were also mined within the corpus to ensure completeness. This search was then complemented by a query in the Web of Science (WoS) database using the same keywords and species names, to retrieve any bibliographic resources not included in the original corpus. Finally, a targeted internet search (via Google) was conducted to identify additional technical and agronomic documents—such as those from USDA, EMBRAPA, INRAE, CIRAD—as well as unpublished reports, master’s theses, or PhD dissertations not indexed in the corpus or in WoS, using the same key words and species name set.

### Sampling and rearing of parasitoids in Guadeloupe

Eggs larvae and pupae of *Diaphania hyalinata* were collected from cucumber fields in Guadeloupe, at the experimental farm Karusmart in December 2023 and August 2024 (INRAE; Petit-Bourg: 16°12.22'N, 61°39.43'W) (Selbonne et al. 2023), and on two commercial market garden farms in August and September 2024 (Petit-Bourg: 16°13.82'N, 61°36.14'W; Abymes: 16°16.83'N, 61°30.74'W). On each site and at each field collection (four in total), *D. hyalinata* were collected in 50 × 50 cm quadrats in cucumbers rows (92 quadrats in total). *Diaphania hyalinata* was collected at the start of the harvest.

After collection, eggs of *Diaphania hyalinata* of each quadrat were transferred in multiple glass tubes (one tube per egg or egg aggregate) with a drop of pure honey to provide food for emerging parasitoids. Larvae collected from a quadrat were placed in a single plastic vial (80 mL); when the number of larvae was too high, larvae from one quadrat were separated into two vials. Cucumber leaves were placed in vials to feed larvae until pupation. Pupae of *D. hyalinata* were placed in separate plastic vials with one plastic vial per quadrat. Specimens were maintained in uncontrolled conditions and monitored each day, until the emergence of larvae (from eggs), moth (from larvae), or adult parasitoids (from eggs, larvae, or pupae of *D. hyalinata*). Parasitoids that emerged were placed in 70% ethanol.

### Morphological identification and imaging of parasitoids

The co-authors of this study include highly skilled taxonomists (JLFT, JYR) with worldwide expertise in both Ichneumonoidea and Chalcidoidea, as well as in Hymenoptera used for biological control in several regions, including the Neotropics.

For all taxa, we applied a consistent and iterative approach to identify species belonging to hyperdiverse and taxonomically difficult genera, including *Apanteles* Förster, 1862 (205 species described from Mesoamerica); *Conura* Spinola, 1837 (260 valid species in the New World (NW)) and *Trichogramma* Westwood, 1833 (108 NW valid species), as well as species from smaller genera such as *Aphanogmus* Thomson, 1858 [very poorly known, ~12 valid NW species, the last revision of new world species dating back to Ashmead (1893)]; *Brachymeria* Westwood, 1829 (53 NW valid species); *Eiphosoma* (61 NW valid species); *Schoenlandella* (4 NW valid species); *Trichospilus* Ferrière, 1930 (9 valid species worldwide).

We first prepared and photographed diagnostic structures to facilitate comparison between our specimens and descriptions or reference material. We then used both monographs and general identification keys to circumscribe the species, or species groups, to which our specimens belong. Subsequently, we examined all original descriptions and relevant subsequent taxonomic treatments to determine which described taxa shared the greatest number of characters with our material. In some cases, identification to a single species was possible; however, in others (e.g., *Trichogramma*), morphological identification was limited to small species groups, or even to cryptic species complexes. We therefore used the most inclusive name when a cryptic species complex been demonstrated (e.g., *Trichogramma* (Pinto et al. 1986)), pending potential confirmation through molecular data, only when expert taxonomists were involved in identifying the sequenced specimens.

Here, we cite the relevant publications used for the identification of *Apanteles* (Muesebeck 1958; Fernández-Triana et al. 2014); *Aphanogmus* (Ferrière 1933; Dessart 1971; Polaszek and La Salle 1995; Salden and Peters 2023); *Brachymeria* (Burks 1960; Bouček and Delvare 1992b); *Conura* (Burks 1940; Bouček and Delvare 1992a; Delvare 1992); *Eiphosoma* (Costa Lima 1953; Fernández-Triana and Ravelo 2007; Onody et al. 2009; Cuéllar-Ramírez et al. 2023; Fernandes et al. 2023); *Schoenlandella* (Dangerfield et al. 1999; Kang et al. 2021); *Trichospilus* (Cherian and Margabandhu 1942; Bouček 1976; Ubaidillah 2006; Narendran 2011; Yang et al. 2015); and *Trichogramma* (Nagaraja et al. 1973; Pinto et al. 1986; Pinto 1998; Querino and Zucchi 2019).

Whenever possible, we compared our specimens with material from reference collections. For instance, *Brachymeria* and *Conura* specimens were compared with material housed in the CBGP collection that had previously been examined against type specimens by Gérard Delvare (CIRAD). Similarly, *Trichogramma* specimens were compared with the CBGP reference collection, largely composed of specimens identified by Bernard Pintureau (INRAE). *Apanteles* specimens were compared with material from the Canadian National Collection of Insects (Ottawa).

This approach does not preclude the possibility of misidentifications, particularly in such challenging groups. Indeed, species of *Trichogramma*, *Apanteles*, and *Aphanogmus* likely comprise complexes of cryptic or very closely related species that may be misinterpreted here. In addition, many undescribed species likely remain in the biogeographical region considered, far exceeding the number of currently described species. Therefore, although the level of uncertainty and potential error remains substantial, it is clearly lower than that observed in current barcoding databases or in ecological studies lacking taxonomic expertise.

Finally, to ensure traceability of our potential errors, all specimens that were non-destructively sequenced or morphologically examined have been mounted, curated, accurately labelled as voucher specimens, and deposited in the CBGP collection, enabling future reassessment or correction.

Images were obtained using a Keyence vHX-5000 multiple-focus imaging system. Assemblage and edition of plates were done using Affinity Photo2 ©. We took photos of the antenna, habitus (lateral view), head (frontal view), mesosoma (dorsal and lateral view), propodeum (dorsal view), and metasoma (dorsal view).

### Barcoding of parasitoids

For each of the eight parasitoid species collected in the field, the 5' end of the cytochrome c oxidase subunit I gene (COI; the “barcode fragment”) was sequenced from one to five specimens. MinION (Oxford Nanopore Technologies) sequencing was carried out for seven of the eight species at Centre de Biologie pour la Gestion des Populations (CBGP) (as part of a larger experiment), while Sanger sequencing was used for the remaining one (*Eiphosoma dentator*) at Institut Sophia Agrobiotech (ISA).

MinION: DNA was extracted from specimens using the Qiagen DNeasy Blood and Tissue Kit following the manufacturer’s protocol with a few modifications detailed in Cruaud et al. 2019. The COI barcode region was amplified using standard primer pairs that were asymmetrically indexed with UMIs, Unique Molecular Identifiers following Hebert et al. (2025). The PCR mix for 1 reaction was composed of 5 µl of Qiagen® Taq Multiplex Mastermix, 2 µl of ultrapure water, 1 µl of forward primer (2 µM), 1 µl of reverse primer (2 µM), 1 µl of DNA. Polymerase Chain Reaction (PCR) was performed using Eppendorf Mastercyclers Nexus under the following conditions: initial denaturation at 95 °C for 15 min; followed by 45 cycles of denaturation at 95 °C for 40 s, annealing at 45 °C for 40 s, and extension at 72 °C for 1 min; with a final extension at 72 °C for 2 min and a hold at 15 °C. Amplicons obtained from all specimens were pooled together with amplicons from a larger, separate experiment. The final pool was purified using AMPure XP beads (Agencourt). End-prep, adapter ligation, and clean-up of the purified pool were performed following the ONT Ligation Sequencing of Amplicons protocol V14 (SQK-LSK114), v. V (ACDE_9163_v114_revV_12Dec2024) for PromethION. The final pool was loaded onto a PromethION flow cell at a concentration of 100 fmol and sequenced on a P2 Solo. Basecalling and FASTQ file generation were performed from POD5 files using Dorado v. 0.9.0 (super-accurate basecalling model v. 4.3.0, 400 bps). After demultiplexing and trimming of UMIs and primers using a custom Python script, reads longer than 600 bp were clustered with VSEARCH v. 2.29.3 (Rognes et al. 2016) using a 0.90 similarity threshold, and a consensus sequence was generated for each cluster. To address contig over-splitting due to polymerase and/or sequencing errors, clusters with consensus sequences showing >98% identity were merged. The reads from these merged clusters were then realigned with MAFFT v. 7.505 (Katoh and Standley 2013) to produce 50% majority-rule ‘meta-consensuses’. Only major (meta)consensus sequences based on more than 100 reads were retained, provided that they were coding, and matched—for their full length—with a congeneric species in the NCBI database with at least 90% identity (as no exact reference sequences were available in NCBI for the sequenced parasitoids). For species for which multiple individuals were sequenced, we also ensured that the same barcode sequence was consistently recovered.

Sanger: for each specimen, a non-destructive DNA extraction was performed with 20 μl of QuickExtract™ DNA Extraction Solution kit (Lucigen® QE09050), during 15 min at 65 °C and 2 min at 98 °C. Then COI was amplified using the primer pair: LCO 1490 (5'-GTCAACAAATCATAAAGATATTGG-3') and HCO 2198 (5'-TAAACTTCAGGGTGACCAAAAAATCA-3') (Folmer et al. 1994). For the PCR reaction (performed in a total volume of 25 μL), 1 μL of DNA was used. PCR was performed with the Multiplex PCR Master Mix QIAGEN (Cat No./ID: 206145) with a final concentration of 3 mM MgCl_2_ and 0.125 μL of each of the two primer solutions (100 μM). PCR conditions were as follows: 95 °C for 15 min, followed by 35 cycles of (i) 94 °C for 30s, (ii) 50 °C for 1 min, and 72 °C for 1 min with a final extension at 72 °C for 10 min. PCR products were shipped to Genewiz (Leipzig, Germany) for Sanger sequencing. The obtained sequences were checked and compared to sequences available in NCBI.

### Descriptive analysis of parasitism rate and success

We defined two categories based on developmental life stage: larvae and pupae were analyzed together, while eggs were analyzed separately. For each category, we analyzed three variables: 1) number of *D. hyalinata* individuals collected in the field (either larvae and pupae or eggs); 2) The developmental outcome of the collected individuals, categorized as either parasitoids that successfully emerged, or individuals that reached the next development stage (moth for larvae and pupae or larvae for eggs) and 3) parasitism assessment and parasitoid identification. Larvae and pupae were grouped together and analyzed separately from eggs to distinguish larval parasitoids from egg parasitoids.

The number of *D. hyalinata* individuals collected in the field was used as the baseline (i.e., 100% of larvae and pupae or 100% of eggs) for each category. For each outcome variable, we calculated the percentage relative to this initial number, indicating either successful host development or total parasitism rate, as well as species-specific parasitism rates. To evaluate parasitism, we also determined the relative abundance of each parasitoid species, expressed as the percentage of individuals of that species among all *D. hyalinata* parasitoids that emerged.

## Results and discussion

### Synopsis of neotropical parasitoid wasps of *Diaphania hyalinata*

**Figure 1.**
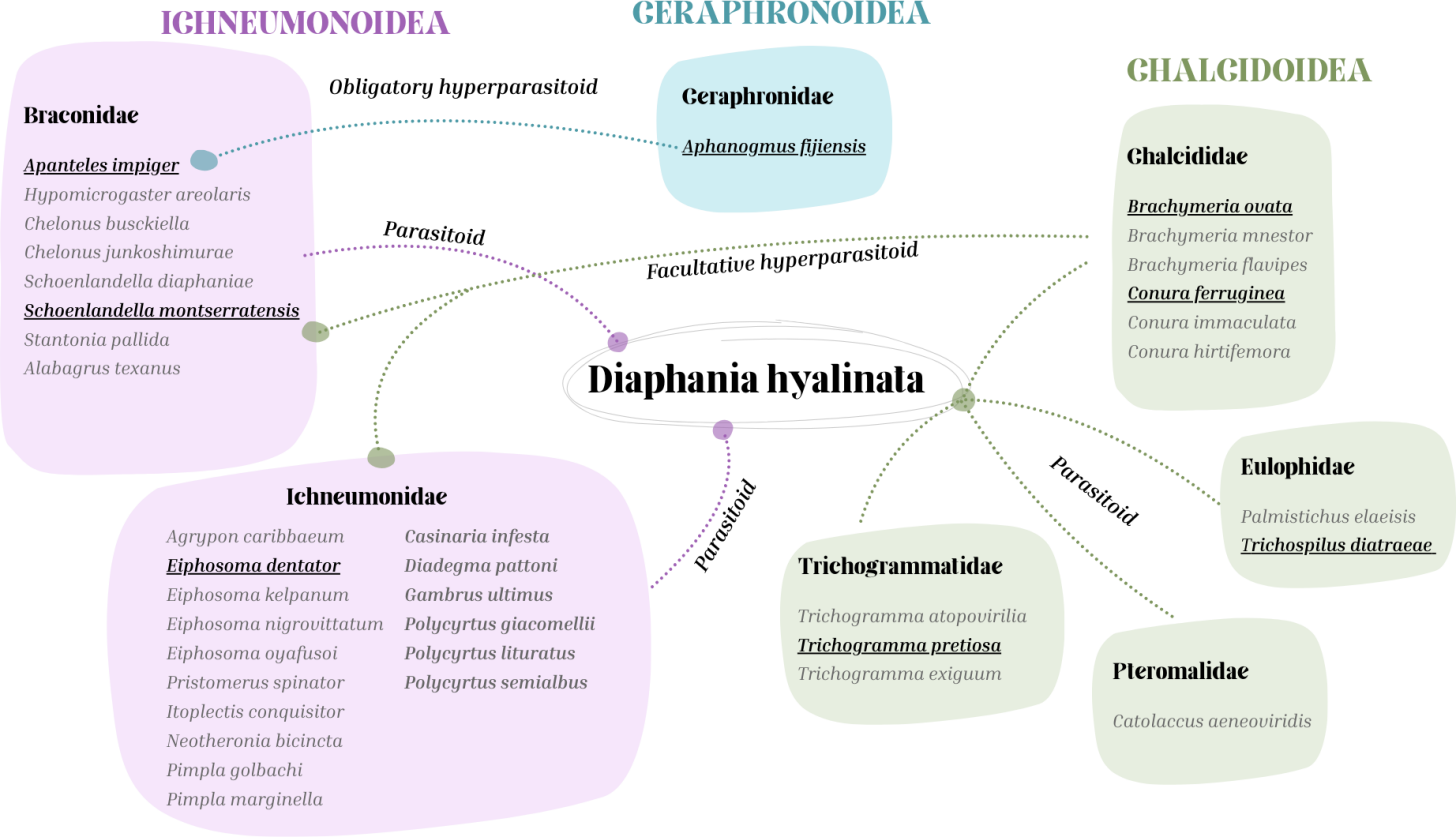
Theoretical host-parasitoid interaction network involving *D. hyalinata* in the neotropics, as reconstructed from our literature review. Species sampled in Guadeloupe during this study are outlined.

This synopsis is based on the analysis of 110 publications, which are cited in the text and fully referenced in the Cited References section of the manuscript. Fig. 1 presents a diagram of the interaction network of parasitoid species associated with *D. hyalinata*, with those collected in Guadeloupe during our field surveys specifically highlighted.

#### Superfamily Ceraphronoidea

**Family Ceraphronidae**

##### Aphanogmus fijiensis

Taxon classificationAnimaliaLepidopteraCeraphronidae

(Ferrière, 1933)

325055AC-C282-587D-9C6F-F87CC67D0B9F

Fig. 2

###### Notes.

This species was collected in our survey. It is cited here for the first time from Guadeloupe, and it is also the first citation of its association with *Diaphania hyalinata* and, likely, *Apanteles impiger*.

**Figure 2.**
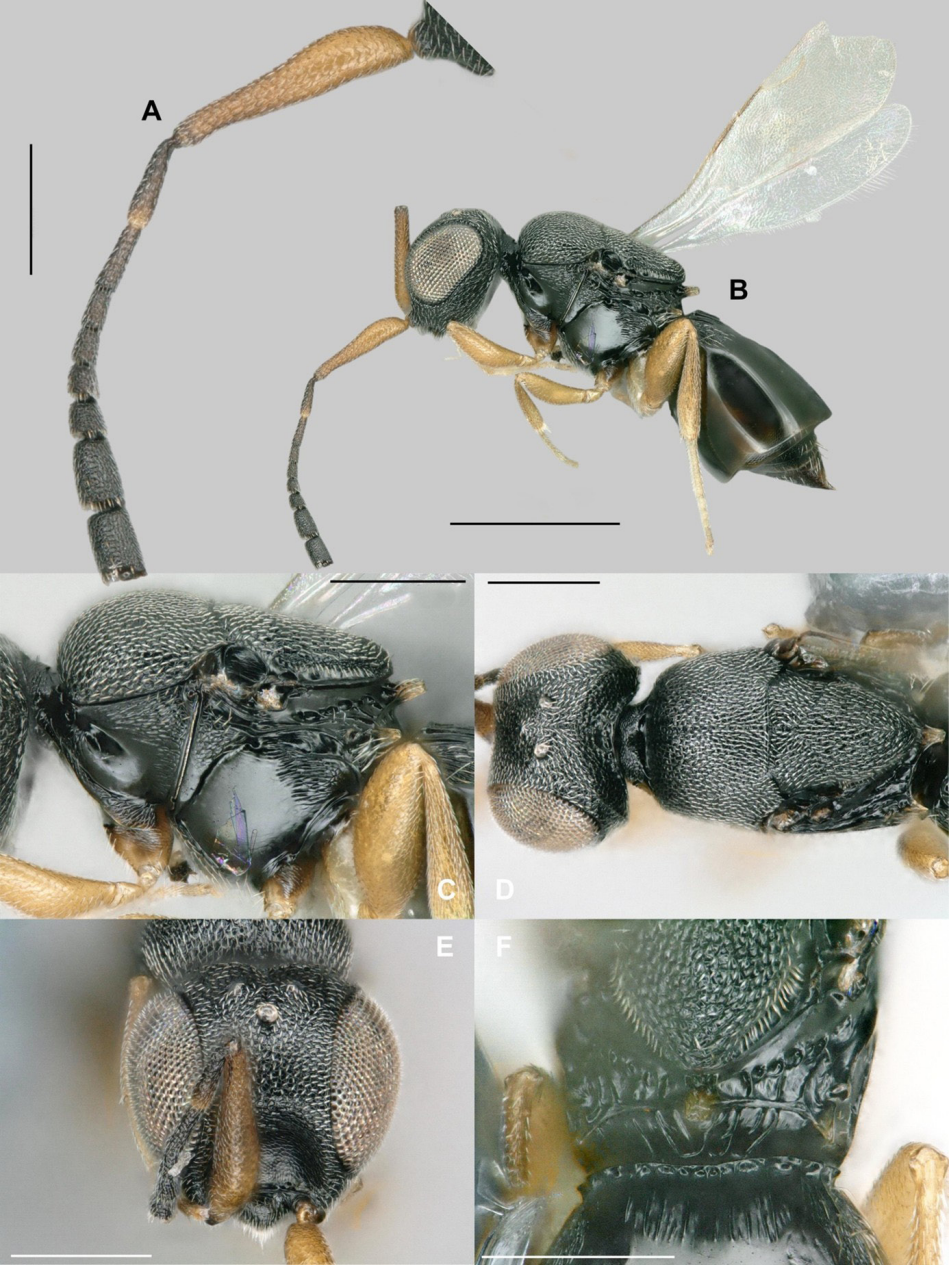
*Aphanogmus fijiensis*, ♀. **A**. Antenna; **B**. Habitus, lateral view; **C**. Mesosoma, lateral view; **D**. Mesosoma, dorsal view; **E**. Head, frontal view; **F**. Propodeum. Scale bars: 200 µm (**A–F**); 500 µm (**B**).

###### Material examined.

Guadeloupe • 2 specimens; Petit–Bourg, INRAE experimental station; 16°12.22'N, 61°39.43'W; 27 Aug 2024; M. Gumbau leg.; reared from field-collected larvae of *Diaphania hyalinata* on cucumber; 1 ♀ analyzed and preserved as voucher (CBGP MGUM0419_0101); NCBI accession PZ362390; other specimens stored in CBGP Cryobox MG-MD-DBF-2024 #2.

###### Distribution.

The species is pantropical and appears to have been introduced in the Neotropics and in Africa with programs of biological control. It is difficult to ascertain the original area of this species, which is known from Australia, India, Fiji, Malaysia, Mauritius, Pakistan, Solomons Islands and Sri Lanka. The species is also reported from Africa without details (Polaszek et al. 1994), cited from Tanzania from where it originated, the population imported in Mauritius (Dessart 1971), and cited from Madagascar (Madl 2015). In the Caribbean region it has been reported from Barbados, Martinique, Saint Vincent and Trinidad (identified by Delvare and Boucek in Cochereau 1991).

###### Biology.

*Aphanogmus fijiensis* is a gregarious secondary endoparasitoid (hyperparasitoid) of primary parasitoids of Lepidoptera larvae and pupae. Females oviposit in the cocoons of their hosts (Braconidae or others) and the ecotoparasitoid larva passes through three instars. The complete life cycle of *A. fijiensis* takes ~13–15 days (Peter and David 1990).

###### Hosts.

*Aphanogmus fijiensis* parasitizes a wide range of primary parasitoids, mostly Braconidae belonging to the diverse genera *Apanteles*, *Cotesia* and *Dolichogenidea*. It also develops within cocoons of *Gonozius sensorius* Gorth (Bethylidae) (Peter and David 1991). This species (under the name *Calliceras fijiensis*) was originally described as a hyperparasitoid of *Tirathaba* sp. (Pyralidae) through *Apanteles tirathabae* Wilkinson, 1928 (Ferrière 1933).

#### Superfamily Chalcidoidea

**Family Chalcididae**

##### Brachymeria ovata

Taxon classificationAnimaliaLepidopteraChalcididae

(Say, 1824)

BB4A6DB9-FFE3-5D0B-A707-F83FEB956691

Fig. 3

###### Notes.

This species was collected in our survey. It is cited here for the first time from Guadeloupe.

**Figure 3.**
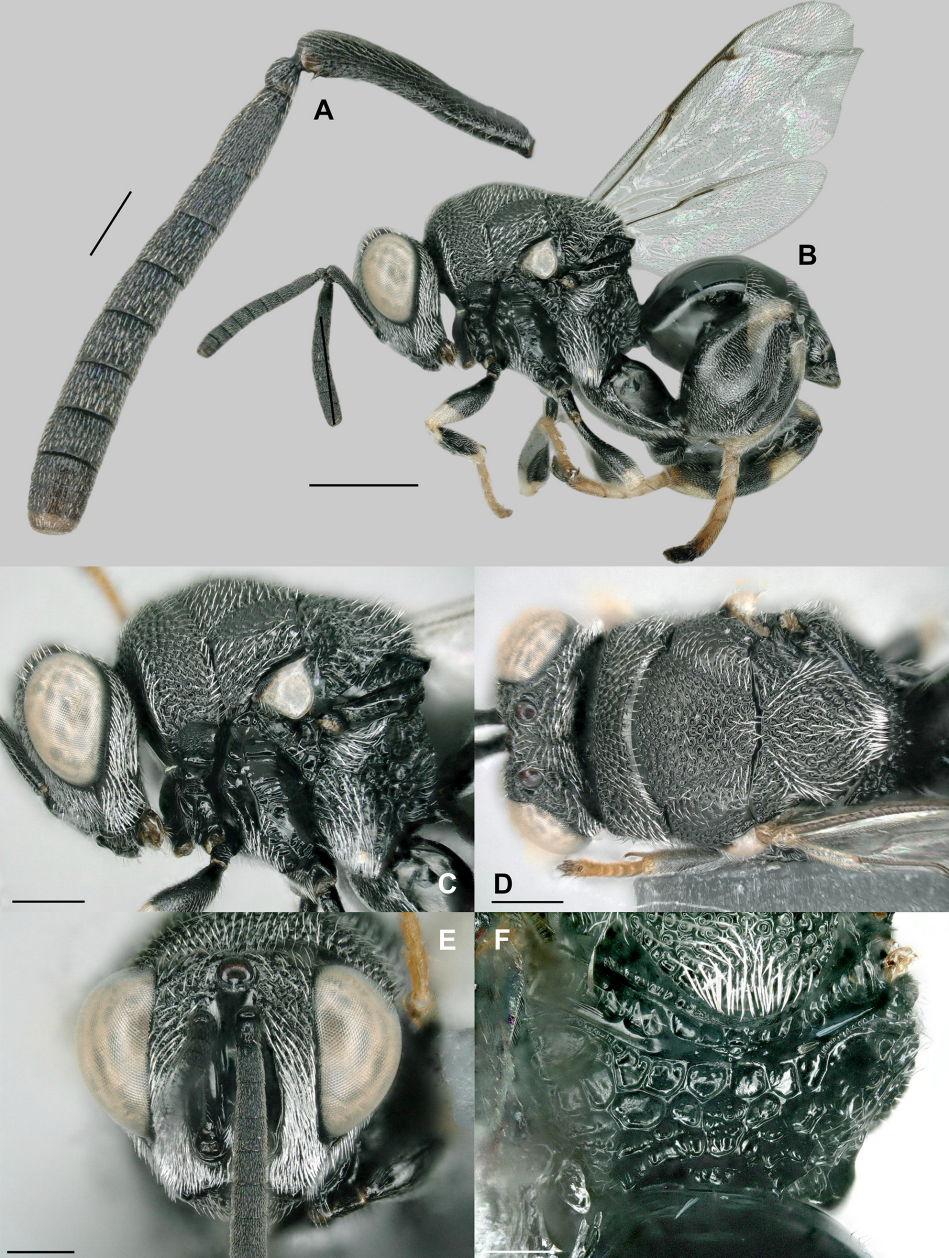
*Brachymeria ovata*, ♀. **A**. Antenna; **B**. Habitus, lateral view; **C**. Mesosoma, lateral view; **D**. Mesosoma, dorsal view; **E**. Head, frontal view; **F**. Propodeum. Scale bars; 100 µm (**A**); 500 µm (**C–F**); 1 mm (**B**).

###### Material examined.

Guadeloupe • 2 ♀; Petit–Bourg, INRAE experimental station; 16°12.22'N, 61°39.43'W; 27 Aug 2024; M. Gumbau leg.; reared from field-collected larvae of *Diaphania hyalinata* on cucumber; (CBGP MGUM0426_0101, MGUM0431_0101) • 1 specimen; Abymes, commercial market garden farm; 16°16.83'N, 61°30.74'W; 6 Sept 2024; M. Gumbau leg.; reared from field-collected larvae of *Diaphania hyalinata* on cucumber; specimens stored in CBGP Cryobox MG-AG24-JF.

###### Distribution.

The species is widespread in the Neotropics and Nearctic, from Canada to Uruguay through meso-America and the Caribbean subregion (Cuba, Grenadines, Puerto Rico, St. Kitts and Nevis).

###### Biology.

*Brachymeria ovata* is a solitary larval parasitoid of Lepidoptera. No information is currently available on the larval morphology, duration of development or number of larval instars. Burks (1960) observed that *B. ovata* larvae do not consume the entire contents of the host and uneaten host tissues remain in the pupa after the adult parasitoid has emerged. Females of *Brachymeria ovata* spend a considerable amount of time examining freshly formed pupae before oviposition (Burks 1960).

###### Hosts.

The species is highly polyphagous with ~100 host species belonging to ≥14 families. *Brachymeria ovata* oviposited and developed in pupae of *Pseudoplusia includens* (Walker), the soybean looper moth; *Trichoplusia ni* (Hübner), the cabbage looper; *Heliothis zea* (Boddie), the corn earworm; *Chloridea virescens* (F.), the tobacco budworm; *Spodoptera exigua* (Hübner), the beet armyworm; *Spodoptera frugiperda* (J. E. Smith), the fall armyworm (all Lepidoptera: Noctuidae of economic importance) and *Anticarsia gemmatalis* (Hübner), the velvetbean caterpillar (Erebidae). *Brachymeria ovata* was first cited from Paraguay associated with *Diaphania hyalinata* (Parker et al., 1953) and then in several other neotropical countries (Peck 1963; Herting 1975). The species can also be an hyperparasitoid on Eupelmidae (De Santis 1967). *Brachymeria ovata* has been considered as a candidate for biological control against several lepidopteran pest species (Rogers and Sullivan 1991).

##### Brachymeria mnestor

Taxon classificationAnimaliaLepidopteraChalcididae

(Walker, 1841)

1F10FACA-AF72-544C-B226-2BE9AE19B0EE

###### Notes.

This species was not collected in our survey.

###### Distribution.

Widely distributed in the Neotropics, Argentina, Brazil, Ecuador, Uruguay, Peru (UCD Community 2023).

###### Biology.

*Brachymeria mnestor* is mostly a solitary idiobiont and pupal parasitoid of Lepidoptera (Gil-Santana and Tavares 2006).

###### Hosts.

*Brachymeria mnestor* is a common species, frequently reared from several species of lepidopteran pupae in the families Crambidae, Ctenuchidae, Hesperiidae, Noctuidae, Pieridae (UCD Community 2023) but also Papilionidae such as *Parides ascanius* (Cramer, 1775) (Tavares et al. 2006) and Nymphalidae (*Actinote parapheles* Jordan, 1913) (Gil-Santana and Tavares 2006). The species was reared from *D. hyalinata* (Linnaeus) which is a primary host of *Brachymeria mnestor* (= *B. comitator*) (De Santis 1979). *Brachymeria mnestor* can also develop as a facultative hyperparasitoid of tachinid flies that primary parasitize Lepidoptera (Tavares et al. 2013).

##### Brachymeria flavipes

Taxon classificationAnimaliaLepidopteraChalcididae

(Fabricius, 1793)

DD90CB97-2691-50B8-B5DD-5D38137E9F9A

###### Notes.

This species was not collected in our survey.

###### Distribution.

*Brachymeria flavipes* mostly occurs in the Caribbean subregion of the Neotropics: British Virgin Islands, Cuba, Dominican Republic, Florida, Jamaica, Mexico, Puerto Rico.

###### Biology.

*Brachymeria flavipes* (under the name *B. robusta*) is an endoparasitoid idiobiont of Lepidoptera pupae (Burks 1960). This polyphagous species has a rather low prevalence on their host (~10%) but can reach 60% parasitism rate in certain conditions (Cassani 1985).

###### Hosts.

*Brachymeria flavipes* is highly polyphagous attacking several pests such as *Alabama argillacea* (Hübner, 1823), the cotton leafworm; *Lymire edwardsii* (Grote, 1881), the rubber tree caterpillar; *Mocis latipes* (Guenée, 1852), the striped grass looper (Erebidae); *Helicoverpa zea* (Boddie, 1850) and *Spodoptera frugiperda* (J. E. Smith, 1797) (both Noctuidae); *Oiketicus kirbyi* (Guilding, 1827), the Argentine bagworm, a key pest of avocado (Psychidae) but also on other lepidoptera families *Hyles lineata* (Fabricius, 1775) (Sphingidae); *Megalopyge krugii* (Dewitz, 1897) (Megalopygidae); *Neonympha* sp. (Nymphalidae) and *Papilio cresphontes* (Cramer, 1777) (Papilionidae). The species was considered a primary parasitoid of *D. hyalinata* by De Santis (1989).

###### Comment.

This species was recognized as *Brachymeria robusta* (Cresson, 1865) in Burks (1960) and was frequently misinterpreted in the last century (Bouček and Delvare 1992a). Therefore, some of the host associations cited in the literature may be wrong and need to be confirmed.

##### Conura ferruginea

Taxon classificationAnimaliaLepidopteraChalcididae

(Fabricius, 1804)

8F6F9C34-477F-56BA-BC5C-909AB047861C

Fig. 4

###### Notes.

This species was collected in our survey. It is cited here for the first time from Guadeloupe.

**Figure 4.**
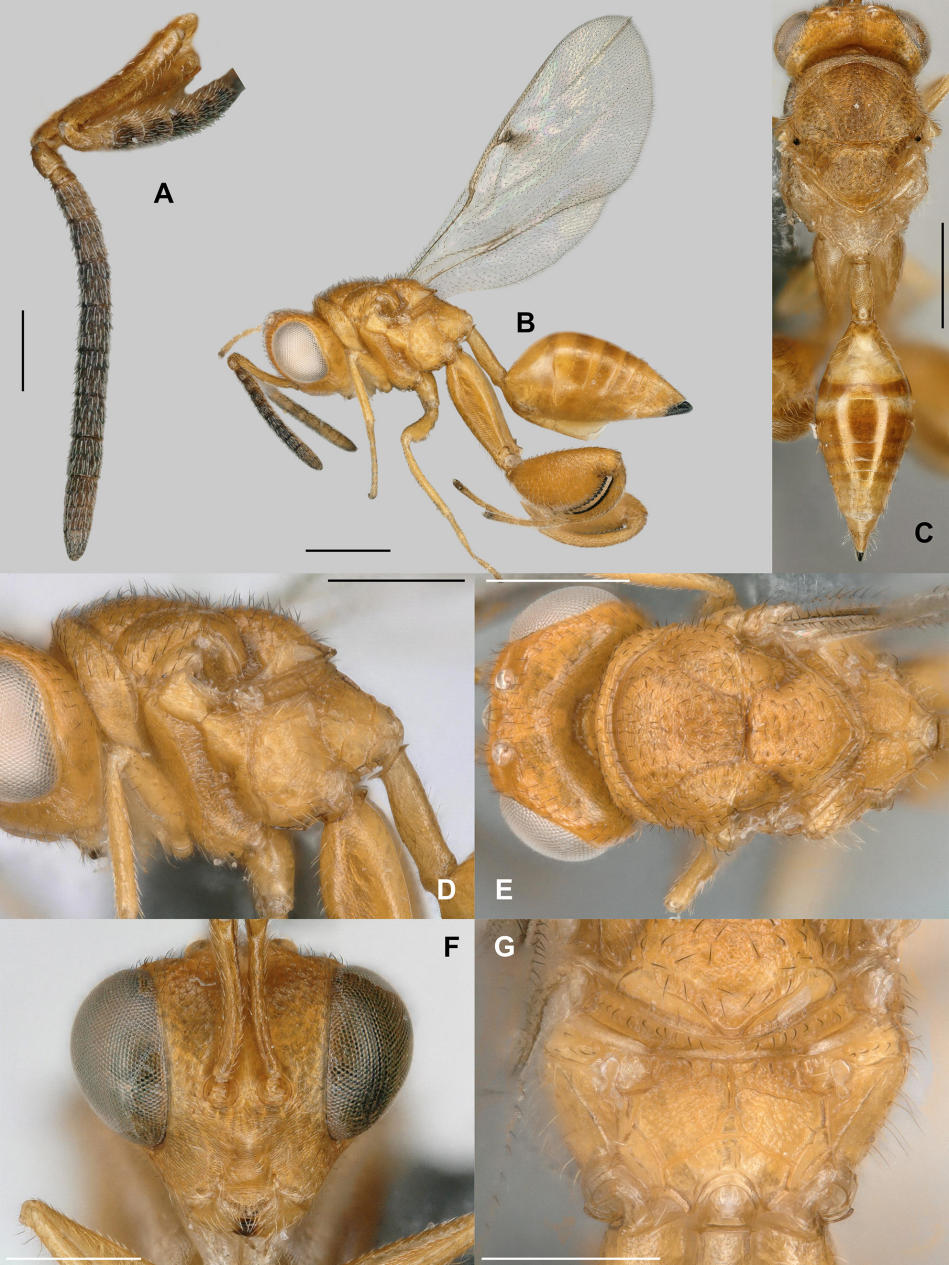
*Conura ferruginea*, ♀. **A**. Antenna; **B**. Habitus, lateral view; **C**. Habitus, dorsal view; **D**. Mesosoma, lateral view; **E**. Mesosoma, dorsal view; **F**. Head, frontal view; **G**. Propodeum. Scale bars; 200 µm (**A, D–G**); 500 µm (**B, C**).

###### Material examined.

Guadeloupe • 2 ♀; Petit–Bourg, INRAE experimental station; 16°12.22'N, 61°39.43'W; 27 Aug 2024; M. Gumbau leg.; reared from field-collected larvae of *Diaphania hyalinata* on cucumber; (CBGP MGUM0425_0101, MGUM0427_0101); NCBI accessions PZ362391–PZ362392. • 4 specimens; Abymes, commercial market garden farm; 16°16.83'N, 61°30.74'W; 6 Sept 2024; M. Gumbau leg.; reared from field-collected larvae of *Diaphania hyalinata* on cucumber; specimens stored in CBGP Cryobox MG-AG24-JF. • 1 specimen; Petit-Bourg, commercial market garden farm; 16°13.82'N, 61°36.14'W; 27 Sept 2024; M. Gumbau leg.; reared from field-collected larvae of *Diaphania hyalinata* on cucumber; specimens stored in CBGP Cryobox MG-AG24-SM.

###### Distribution.

The species is widespread in the Neotropical region, occurring in Brazil, Colombia, Costa Rica, Ecuador, Guyana, and is also present in the Caribbean subregion (Saint Vincent and the Grenadines, Trinidad and Tobago).

###### Biology.

Very little is known about the biology of *Conura ferruginea*, and the species have rarely been reared. It is possibly a primary endoparasitoid of Lepidoptera pupae, potentially acting as a facultative hyperparasitoid.

###### Hosts.

The species (previously named *Spilochalcis fulvescens*) was obtained from pupae of *D. hyalinata* in Cuba (Thompson 1955; De Santis 1979). Myers (1931) stated that *C. ferruginea* emerged from *Diaphania*, but later, he found that there was a species of Tachinidae attacking the pupa of *Diaphania* as a primary host (Myers 1932).

##### Conura immaculata

Taxon classificationAnimaliaLepidopteraChalcididae

(Cresson, 1865)

E8AAADA6-E2E1-5D8E-ADFB-AC6BB488D6F4

###### Notes.

This species was not collected in our survey

###### Distribution.

*Conura immaculata* is widely distributed in the Neotropics occurring from southern USA and Mexico to Brazil and Paraguay. The species is also present in the Caribbean subregion (Cuba, Dominican Republic, Trinidad and Tobago).

###### Biology.

The species is an idiobiont endoparasitoid of Lepidoptera larvae and pupae, but is often a facultative hyperparasitoid (Bouček and Delvare 1992a). The species displays strong size variation depending on the size of its host. When reared from larger hosts, such as Lepidoptera or ichneumonids, both males and females emerge and are relatively large. Conversely, parasitism of smaller hosts, including primary parasitoids such as braconids (*Cotesia*), results in the emergence of small males, with rare females. Host size also influences the sculpture of the mesoscutum and the relative length of scape compared to the flagellum.

###### Hosts.

The species is mostly a parasitoid of Lepidoptera attacking a wide range of species across multiple families (Dalceridae, Depressariidae, Hesperiidae, Limacodidae, Noctuidae, Nymphalidae, Oecophoridae, Crambidae, Tineidae). However, it is often found as a facultative hyperparasitoid through Braconidae (*Apanteles*, *Cotesia*, *Meteorus*) and Ichneumonidae (*Campoletis*, *Casinaria*). *Conura immaculata* has been reared from *Diaphania hyalinata* in the Dominican Republic (De Santis 1989). As such, the species holds potential economic value as a biological control agent.

##### Conura hirtifemora

Taxon classificationAnimaliaLepidopteraChalcididae

(Ashmead, 1885)

DE3261B6-0FF0-573E-A30E-06CA46BB20B6

###### Notes.

This species was not collected in our survey.

###### Distribution.

The species is widespread throughout the neotropics, ranging from Alaska to southern Brazil, and is also present in the Caribbean subregion (Cuba, Dominican Republic, Haiti, Leeward islands, Puerto Rico, Trinidad and Tobago).

###### Biology.

*Conura hirtifemora* is an idiobiont endoparasitoid of Lepidoptera and Diptera pupae, and may preferentially act as a facultative hyperparasitoid of their primary parasitoids. Nothing is known on its life-cycle or larval morphology (Maes 1999).

###### Hosts.

The species parasitizes various small Lepidoptera species but is frequently reared from syrphid larvae (Diptera), including those of *Mesogramma*, *Mesograpta*, *Platychirus* and *Toxomerus*. *Conura hirtifemora* is also a commonly encountered as a facultative hyperparasitoid via braconids (*Apanteles*) or Ichneumonids (*Diadegma*). *Conura hirtifemora* has been reared from *Diaphania hyalinata* (De Santis 1979).

##### Conura side

Taxon classificationAnimaliaLepidopteraChalcididae

(Walker, 1843)

10E0092B-3155-542A-BCDE-9C82DB05FC07

###### Notes.

This species was not collected in our survey.

###### Distribution.

The species is common and widespread in the Nearctic region and in the Mesoamerican subregion. In the Caribbean subregion, the species is reported from Cuba and the Dominican Republic.

###### Biology.

*Conura side* is an idiobiont endoparasitoid of Lepidoptera pupae. Females mate after a short nuptial courtship, oviposition occurs 6–8 days after copulation and 1–4 eggs are laid in each host, but only one individual emerges. Adults emerge from 20–25 days after oviposition at ~ 22 °C, and they live up to five months. Schaffner (1959) observed that *S. side* had at least two generations per year (Arthur 1958; McNeil and Rabb 1973).

###### Hosts.

*Conura side* is also a polyphagous species associated with >50 species of Lepidoptera, several of which are notable pests, and belonging to ~20 families (McNeil and Rabb 1973; UCD Community 2023). The species has been cited (under the name *Spilochalcis flavopicta*) as a parasitoid of *D. hyalinata* in Dominican Republica and Argentina (De Santis 1989). The species also parasitizes Diptera (Agromyzidae) and Coleoptera (mostly Curculionidae and Chrysomelidae). *Conura side* has been released for biological control of *Cassida rubiginosa* Muller, a chrysomelid considered an invasive pest and defoliator of some *Carduus* species (Tipping 1993). The species is also frequently cited as a facultative hyperparasitoid on Braconidae (*Apanteles*, *Meteorus*) and Ichneumonidae (*Campoletis* and *Diadegma*), among others (Herting 1975; Lee and Heimpel 2005).

#### Family Eulophidae

##### Palmistichus elaeisis

Taxon classificationAnimaliaLepidopteraEulophidae

(Delvare & LaSalle, 1993)

21C7AEE2-92A9-5C67-8E4B-700E339232C9

###### Notes.

This species was not collected in our survey.

###### Distribution.

Widespread in the Neotropics from Costa Rica to Brazil, Colombia, and Ecuador

###### Biology.

*Palmistichus elaeisis* is a gregarious endoparasitoid, mostly of Lepidoptera pupae. The duration of its life cycle depends on the host species and ranges from 21 days when reared on *Anticarsia gemmatalis* Hübner, 1818 (Erebidae) to 24 days when reared on *Bombyx mori* (L., 1758) (Bombycidae), possibly due to differences in nutritional resources between hosts. The number of individuals emerging from a single pupa also varies, from ~100 in smaller hosts up to 500 in larger hosts such as *Bombyx mori* (Pereira et al. 2010). *Palmistichus. elaeisis* exhibits superparasitism, particularly self-superparasitism. Increased parasitoid oviposition favored the suppression of the host immune response in melonworm pupae, likely due to the injection of symbiotic viruses, teratocytes, and/or ovarian proteins (de S Pereira et al. 2017).

###### Hosts.

*Palmistichus elaeisis* is a polyphagous idiobiont parasitoid of pupae of multiple lepidopteran hosts. It can also develop on larvae of *Hispoleptis subfasciata* (Pic, 1938) (Chrysomelidae) and *Tenebrio molitor* L., 1758 (Morais et al. 2019). Although the species has not been found associated with *D. hyalinata* under field conditions, it has been successfully reared on this host in laboratory (Morais et al. 2019), suggesting potential economic importance as a biocontrol agent against this species.

##### Trichospilus diatraeae

Taxon classificationAnimaliaLepidopteraEulophidae

(Cherian & Margabandhu, 1942)

E97F7640-8914-5915-8C4E-231238B7234B

Fig. 5

###### Notes.

This species was collected in our survey. It is cited here for the first time from Guadeloupe.

**Figure 5.**
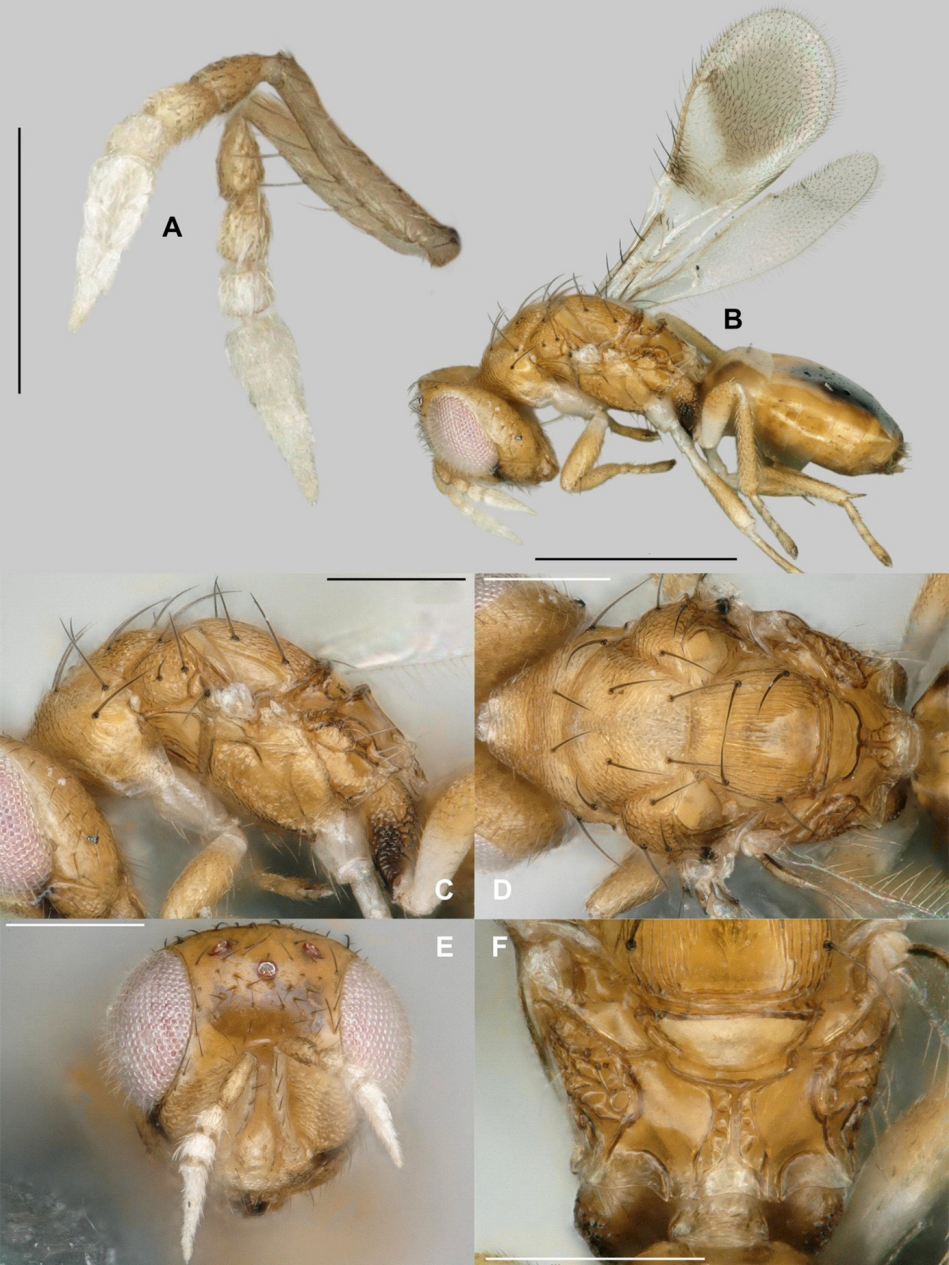
*Trichospilus diatraeae*, ♀. **A**. Antenna; **B**. Habitus, lateral view; **C**. Mesosoma, lateral view; **D**. Mesosoma, dorsal view; **E**. Head, frontal view; **F**. Propodeum. Scale bars: 200 µm (**A, C–F**); 500 µm (**B**).

###### Material examined.

Guadeloupe • 3 ♀; Petit–Bourg, INRAE experimental station; 16°12.22'N, 61°39.43'W; 27 Aug 2024; M. Gumbau leg.; reared from field-collected larvae of *Diaphania hyalinata* on cucumber; 2 ♀ analyzed and preserved as voucher (CBGP MGUM0429_0101, MGUM0430_0101); other specimens stored in CBGP Cryobox MG-MD-DBF-2024 #2.

###### Distribution.

*Trichospilus* Ferrière, 1930, is an Oriental genus of Eulophinae, comprising nine described species. *Trichospilus diatraeae* occurs naturally from India to Papua New Guinea, and have been introduced in multiple countries for biological control programs. In 1963, the species was shipped from India to the USA and to Trinidad for laboratory trials against *Diatraea* spp. which are serious pests of sugarcane in the neotropics (Bennett et al. 1987). It now occurs widely in the Western Hemisphere from Florida (USA) and Mexico to Brazil (where it was not intentionally introduced) and has been cited from the Caribbean subregion (Puerto Rico, and Trinidad and Tobago).

###### Biology.

The species is primarily an idiobiont gregarious pupal parasitoid of gramineous stem borers (Lepidoptera). The number of individuals emerging from the host depends on its size but can each ~1500 individuals per pupa in some large moths (*Spodoptera cosmioides* Walker, 1858). The egg-to-adult development cycle of *T. diatraeae* is ~1 month long (Zaché et al. 2012).

###### Hosts.

*Trichospilus diatraeae* is highly polyphagous being reported from a large variety of hosts (> 50 species of Lepidoptera, in 12 families), among them several agricultural pests (UCD Community 2023). The species was reported parasitizing *D. hyalinata* in Brazil by Melo et al. (2011) and da Silva et al. (2015).

#### Family Pteromalidae

***Catolaccus aeneoviridis*** (Girault) has also been cited as a parasitoid of *D. hyalinata* (Barba et al. 2004); however, this record is doubtful as species of *Catolaccus* are primarily known to parasitize Curculionidae (Gibson 2013).

**Notes**. This species was not collected in our survey.

#### Family Trichogrammatidae

##### Trichogramma atopovirilia

Taxon classificationAnimaliaLepidopteraTrichogrammatidae

(Oatman & Platner, 1983)

4C6A0A3D-8D50-5089-B0D1-8D7A5430316D

###### Notes.

This species was not collected in our survey.

###### Distribution.

The species is widely distributed in Meso- and South America ranging from Texas and Mexico to Brazil. The species also occurs in the Caribbean subregion (Cuba, Jamaica, Dominican Republic, Puerto Rico).

###### Biology.

Like all other *Trichogramma* species, *T. atopovirilia* parasitizes eggs of Lepidoptera. Females insert their ovipositor through the eggshell to lay one egg inside. The developing larva consumes the contents of the host egg, killing the embryo. The full life cycle, from egg to adult, is rapid and can be completed in 7–10 days under optimal conditions (Melo et al. 2007).

###### Hosts.

This species has been recorded from ~20 species of Lepidoptera (UCD Community 2023), some of economic importance (Zucchi et al. 2010). It was reared from eggs of *D. hyalinata* in Brazil (Polanczyk et al. 2009). This species has been reared for biological control of *Spodoptera frugiperda* (Smith, 1797) (Noctuidae).

##### Trichogramma pretiosa

Taxon classificationAnimaliaLepidopteraTrichogrammatidae

(Riley, 1879)

663F9234-D84F-5A85-914C-07071300AFDA

Fig. 6

###### Notes.

This species was collected during our survey. It is officially cited here for the first time from Guadeloupe, although its presence had previously been mentioned through a personal communication by Jean Etienne, as reported in Dumbardon-Martial et al. (2018).

**Figure 6.**
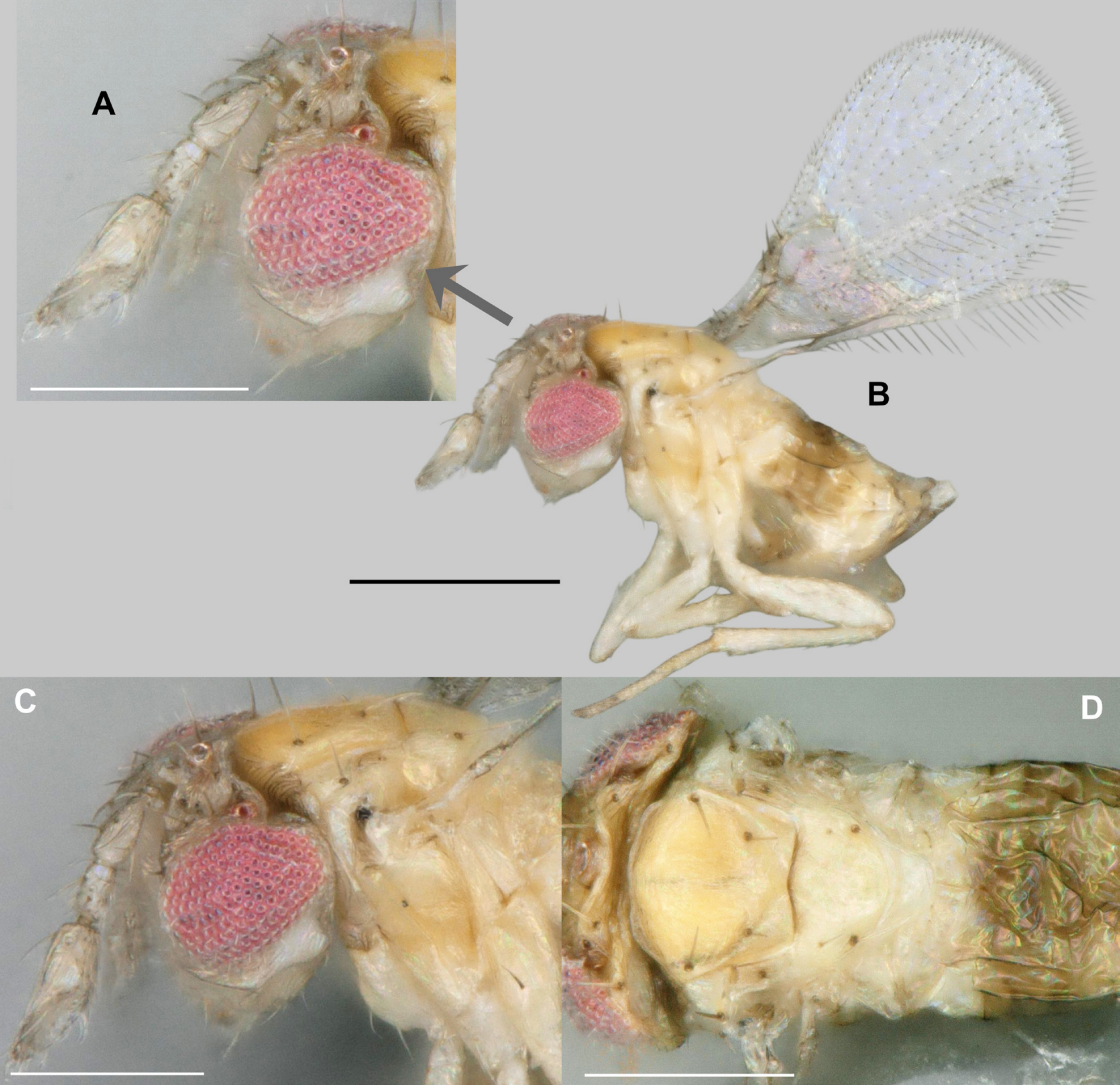
*Trichogramma pretiosa*, ♀. **A**. Antenna and head, lateral view; **B**. Habitus, lateral view; **C**. Mesosoma, dorsal view; **D**. Mesosoma, lateral view. Scale bars; 100 µm (**A, C, D**); 200 µm (**B**).

###### Material examined.

Guadeloupe • 108 specimens; Petit–Bourg, INRAE experimental station; 16°12.22'N, 61°39.43'W; 6 Dec 2023; M. Gumbau leg.; reared from field-collected eggs of *Diaphania hyalinata* on cucumber; specimens stored in ISA Cryobox MG-DBF23-ELEVAGE • 2 specimens; same collection data as for preceding; 27 Aug 2024; 1 ♀ analyzed and preserved as voucher (CBGP MGUM0416_0101); NCBI accessions PZ362393; other specimens stored in CBGP Cryobox MG-MD-DBF-2024 #2. • 3 specimens; Petit-Bourg, commercial market garden farm; 16°13.82'N, 61°36.14'W; 27 Sept 2024; M. Gumbau leg.; reared from field-collected larvae of *Diaphania hyalinata* on cucumber; specimens stored in CBGP Cryobox MG-AG24-SM.

###### Distribution.

*Trichogramma pretiosa* is widely distributed in the Nearctic and Neotropical regions ranging from Canada to Chile, and occurring in the Caribbean subregion including Martinique, Virgin Islands, Cuba, and Dominican Republic.

###### Biology.

Similar to that described for *T. atopovirilia*.

###### Hosts.

*Trichogramma pretiosa* is one of the most polyphagous species of the genus, cited from tens of host species. It is one of the most widely used species of *Trichogramma* for the biological control of agricultural pests (Zucchi et al. 2010). In the Americas, it has been successfully used against several lepidopteran pests. *Diaphania hyalinata* is cited as a primary host of *T. pretiosa* (Letourneau 1987; Pinto 1998; Polanczyk et al. 2009, 2011; Dumbardon-Martial et al. 2018).

###### Comment.

According to the rules of zoological nomenclature (International Commission on Zoological Nomenclature (ICZN 1999), the specific epithet must agree in gender with the genus name when it is an adjective. *Trichogramma* is a neuter noun (derived from the Greek *gramma*, meaning “written”), so an adjectival epithet should be in the neuter form, pretiosum and not pretiosa. However, if the original author used pretiosa as a noun in apposition (for example, a substantive), it remains invariable, regardless of the gender of the genus name. Consequently, unless there is historical evidence that pretiosa was intentionally used as an adjective, which is not the case, pretiosa should be treated as a noun in apposition. Therefore, gender agreement with the neuter genus should not be applied and the original form pretiosa should be retained (UCD Community 2023).

##### Trichogramma exiguum

Taxon classificationAnimaliaLepidopteraTrichogrammatidae

(Pinto & Platner, 1978)

45441D42-F509-5CE6-AE26-246A4DFAF04C

###### Notes.

This species was not collected in our survey.

###### Distribution.

The species is widely distributed throughout the Nearctic and Neotropical regions, ranging from Canada to Chile, and is present in the Caribbean subregion, including Barbados, Bermuda, Cuba, Grenada, the Grenadines, Jamaica, St. Kitts and Nevis, and Trinidad and Tobago (UCD Community 2023).

###### Biology.

Similar to that described for *T. atopovirilia*.

###### Hosts.

*Trichogramma exiguum* is another polyphagous species of *Trichogramma*, and the species has been recorded from eggs of > 40 species of Lepidoptera belonging to ten families (UCD Community 2023), among them *D. hyalinata* (Polanczyk et al. 2011).

#### Superfamily Ichneumonoidea

**Family Braconidae**

**Subfamily Microgastrinae**

##### Apanteles impiger

Taxon classificationAnimaliaLepidopteraBraconidae

Muesebeck, 1958

730B5908-6FC0-53AA-859D-2F300582A5C9

Fig. 7

###### Notes.

This species was collected in our survey. It is cited here for the first time from Guadeloupe.

**Figure 7.**
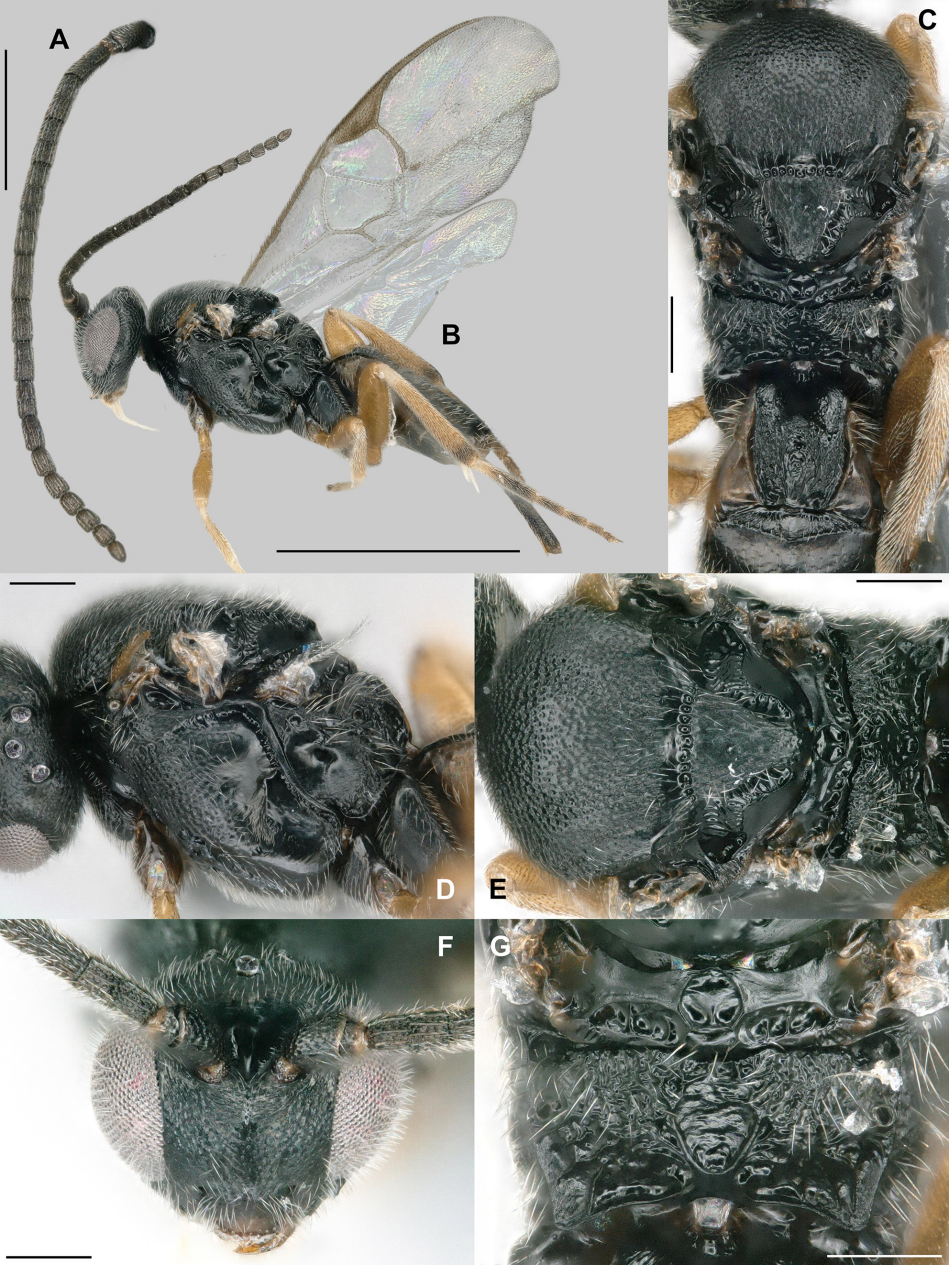
*Apanteles impiger*, ♀. **A**. Antenna; **B**. Habitus, lateral view; **C**. Mesosoma and metasoma, dorsal view; **D**. Mesosoma, lateral view; **E**. Mesosoma, dorsal view; **F**. Head, frontal view; **G**. Propodeum. Scale bars; 200 µm (**A, C–G**); 500 µm (**B**).

###### Material examined.

Guadeloupe • 2 ♀; Petit–Bourg, INRAE experimental station; 16°12.22'N, 61°39.43'W; 27 Aug 2024; M. Gumbau leg.; reared from field-collected larvae of *Diaphania hyalinata* on cucumber; (CBGP MGUM0423_0101, MGUM0424_0101); NCBI accessions PZ362382–PZ362383.

###### Distribution.

Cuba and Puerto Rico (Muesebeck 1958). Cave (1995) and Rodríguez and Flores (2007) cited the species from Honduras and Nicaragua, respectively, but the correct identification of the wasps cannot be confirmed and remains doubtful.

###### Biology.

The species is a solitary koinobiont endoparasitoid of *Diaphania hyalinata* in both countries (Muesebeck 1958). As in documented *Apanteles*, the female oviposits in early instars of the host larva. Upon completing its development, the parasitoid larva emerges from the fourth or fifth instar of the host. After emergence, the larva spins a white cocoon in which it pupates. The adult emerges approximately six or seven days later (Rodríguez and Flores 2007).

###### Hosts.

Only associated with *D. hyalinata* (Crambidae).

###### Comment.

An unidentified species of *Apanteles* was cited from *D. hyalinata* L. in Cuba by Thompson (1953).

##### Hypomicrogaster areolaris

Taxon classificationAnimaliaLepidopteraBraconidae

(Blanchard, 1947)

FDCC1E3F-A5BE-5302-8997-F3E095CECDE0

###### Notes.

This species was not collected in our survey.

###### Distribution.

The majority of the 49 species of *Hypomicrogaster* Ashmead, 1898 occurs in the Neotropics (Fernandez-Triana et al. 2020). *Hypomicrogaster areolaris*, also cited as *H. diaphaniae* Muesebeck, 1968, is widespread in the Western Hemisphere from the USA and Mexico to Central America (Belize, Costa Rica, El Salvador, Jamaica, Panama) and South America (Argentina, Brazil, Ecuador, Uruguay).

###### Biology.

*Hypomicrogaster* species are gregarious koindobiont endoparasitoids. The female lays multiple eggs at one time into a single host (Shaw 1995). The gregarious larvae develop internally and, upon emergence, spin their cocoons near the host’s corpse, from which the adults subsequently emerge.

###### Hosts.

*Hypomicrogaster areolaris* has been reared from *D. hyalinata* feeding on zucchini plants in Costa Rica as well as from an unidentified *Diaphania* feeding on *Tabernaemontana* (= *Stemmadenia*) *robinsonii* (Woodson) (Apocynaceae) (Valerio and Whitfield 2015). The species is also reported to develop in Gelechiidae. In total, species of *Hypomicrogaster* are associated with ~10 different families of Lepidoptera in the Neotropics (Whitfield et al. 2018).

###### Comment.

An unidentified species of *Hypomicrogaster* was imported from Colombia in 1991 by J. L. Capinera and release against *Diaphania hyalinata* and *D. nitidalis* in Florida (Frank and McCoy 1993).

#### Subfamily Cheloninae

##### Chelonus busckiella

Taxon classificationAnimaliaLepidopteraBraconidae

(Viereck, 1912)

7340B07E-96D8-5FF9-AAE4-3CDDF71B5265

###### Notes.

This species was not collected in our survey.

###### Distribution.

The species was originally described from Montserrat (Viereck 1912) but has also been reported from Brazil and Mexico (González-Hernández 2004).

###### Biology.

The biology of this species is still unknown, but *Chelonus* species are solitary koinobiont egg-larval endoparasitoids of concealed Lepidoptera.

###### Hosts.

Although information on *C. busckiella* is limited, available data suggest that its host range is restricted to Crambidae, including species of *Diaphania*. In Brazil, the species parasitizes *Loxomorpha cambogialis* (Guenée, 1854) (Costa Lima 1962) (Crambidae). *Chelonus busckiella* was collected in Colombia and later introduced into Florida by J. L. Capinera for biological control against *Diaphania hyalinata* and *D. nitidalis* (Frank and McCoy 1993).

##### Chelonus junkoshimurae

Taxon classificationAnimaliaLepidopteraBraconidae

(Sharkey, 2021)

A596D077-FF16-5EC2-AEBA-5C875B1AB1BE

###### Notes.

This species was not collected in our survey.

###### Distribution.

Only known from Costa Rica.

###### Biology.

Biology unknown, the species could also be a solitary koinobiont egg-larval endoparasitoid.

###### Hosts.

*Chelonus junkoshimurae* has only been reared from *Diaphania hyalinata* feeding on *Gurania makoyana* (Cucurbitaceae) (Sharkey et al. 2021).

#### Subfamily Cardiochilinae

##### Schoenlandella diaphaniae

Taxon classificationAnimaliaLepidopteraBraconidae

(Marsh, 1986)

00DE2F42-63D8-5E11-BE13-23EC1D0572D3

###### Notes.

This species was not collected in our survey.

###### Distribution.

Neotropical region (Colombia, Venezuela) and Caribbean subregion (Trinidad and Tobago).

###### Biology.

*Schoenlandella diaphaniae* (also studied under the name *Cardiochiles diaphaniae*) is a solitary endoparasitoid of *Diaphania* larvae (Smith et al. 1994) and exhibits biological characteristics consistent with those of other species in the genus *Schoenlandella*. Female wasps deposit a single egg into *Diaphania* larva. Larval development on the melonworm (*D. hyalinata*) takes ~11 days, although the duration varies significantly depending on host’s age at the time of parasitism. Development on the pickleworm (*D. nitidalis*) was one day shorter (Smith et al. 1994). *Schoenlandella diaphaniae* remained in the pupal stage for 8 to 10 days. The duration between cocoon formation and adult emergence increased slightly with host age at the time of parasitism.

###### Hosts.

Neotropical species of *Schoenlandella* are primarily associated with Crambidae and Noctuidae (Lepidoptera), including several notable agricultural pests such as *Crocidolomia pavonana* (Fabricius, 1794), the cabbage cluster caterpillar (Crambidae), or *Helicoverpa armigera* (Hübner, 1808) (Noctuidae). *Schoenlandella diaphaniae* is associated with *D. hyalinata* and *D. nitidalis*. *Schoenlandella diaphaniae* was collected in Colombia and released multiple times in Florida and Puerto Rico as part of biological control programs targeting *Diaphania* spp. but it does not appear to have established in these regions (Frank and McCoy 1993).

##### Schoenlandella montserratensis

Taxon classificationAnimaliaLepidopteraBraconidae

(Kang, 2021)

5CB9660B-C738-5AFF-9532-0063267886AA

Fig. 8

###### Notes.

This species was collected in our survey. We confirmed this association by rearing *S. montserratensis* from *D. hyalinata* larvae. *Schoenlandella montserratensis* is cited here for the first time from Guadeloupe.

**Figure 8.**
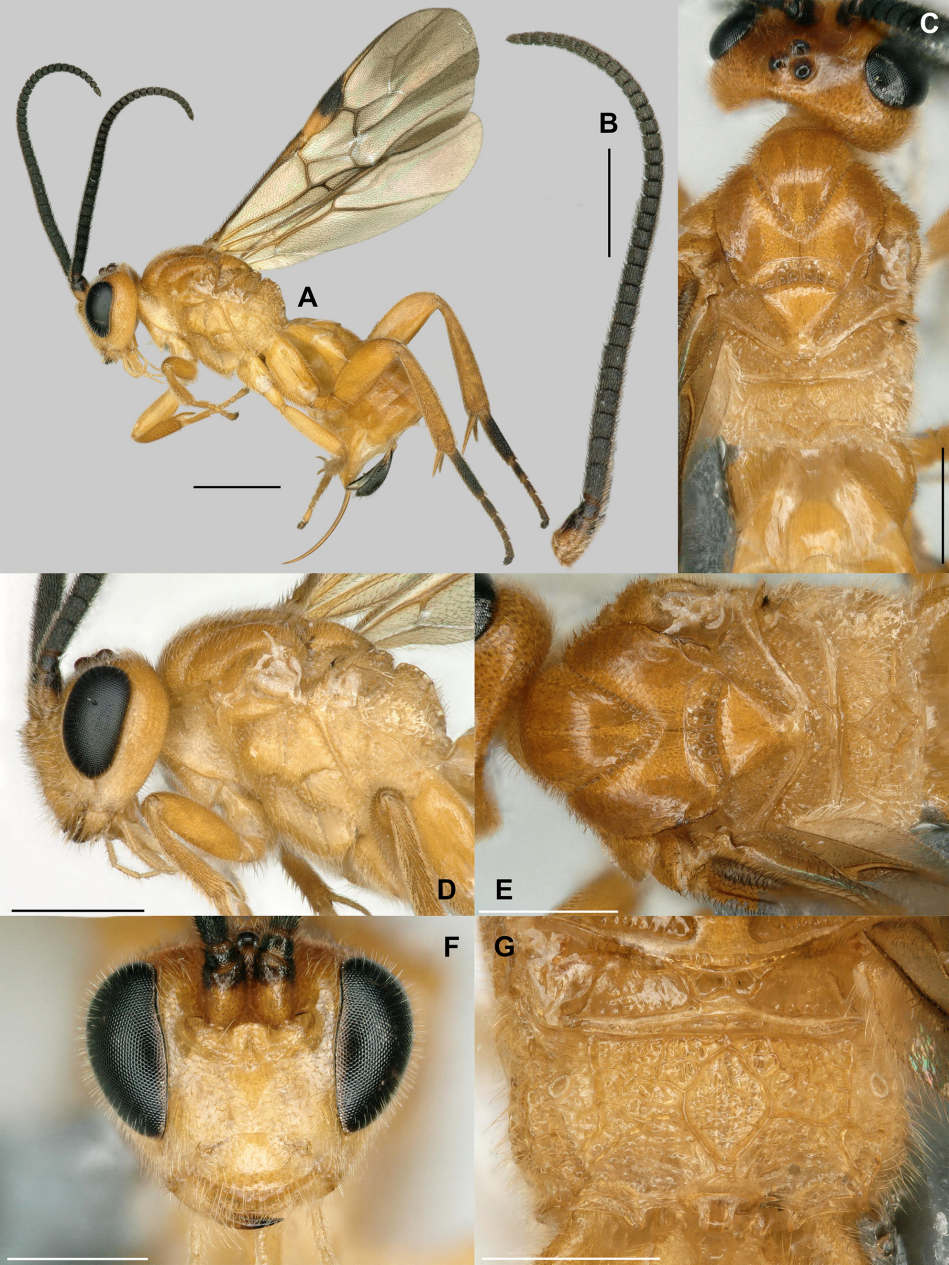
*Schoenlandella montserratensis*, ♀. **A**. Habitus, lateral view; **B**. Antenna; **C**. Mesosoma and metasoma, dorsal view; **D**. Mesosoma, lateral view; **E**. Mesosoma, dorsal view; **F**. Head, frontal view; **G**. Propodeum. Scale bars: 500 µm (**B–G**); 1 mm (**A**).

###### Material examined.

Guadeloupe • 17 specimens; Petit–Bourg, INRAE experimental station; 16°12.22'N, 61°39.43'W; 6 Dec 2023; M. Gumbau leg.; reared from field-collected larvae of *Diaphania hyalinata* on cucumber; specimens stored in ISA Cryobox MG-DBF23-ELEVAGE • 25 specimens; same collection data as for preceding; 27 Aug 2024; 5 ♀ analyzed and preserved as voucher (CBGP MGUM0415_0101, MGUM0417_0101, MGUM0418_0101, MGUM0421_0101, MGUM0422_0101); NCBI accessions PZ362386–PZ362389; other specimens stored in CBGP Cryobox MG-MD-DBF-2024 #2. • 21 specimens; Abymes, commercial market garden farm; 16°16.83'N, 61°30.74'W; 6 Sept 2024; M. Gumbau leg.; reared from field-collected larvae of *Diaphania hyalinata* on cucumber; specimens stored in CBGP Cryobox MG-AG24-JF. • 13 specimens; Petit-Bourg, commercial market garden farm; 16°13.82'N, 61°36.14'W; 27 Sept 2024; M. Gumbau leg.; reared from field-collected larvae of *Diaphania hyalinata* on cucumber; specimens stored in CBGP Cryobox MG-AG24-SM.

###### Distribution.

Caribbean subregion, Montserrat and Guadeloupe.

###### Biology.

Very little is known about the exact biology of this species, but the species appears to be a solitary endoparasitoid of *D. hyalinata* larvae.

###### Hosts.

Female adults of *S. montserratensis* were first collected in a bitter gourd field in Montserrat in 2019. Among the four lepidopteran species known to develop on this host plant, *Diaphania hyalinata* was suspected to be its host (Kang et al. 2021).

#### Subfamily Orgilinae

##### Stantonia pallida

Taxon classificationAnimaliaLepidopteraBraconidae

(Ashmead, 1894)

E0799014-5F68-5387-9E80-2D6B5E82C45D

###### Notes.

This species was not collected in our survey.

###### Distribution.

*Stantonia pallida* is broadly distributed in the Western Hemisphere, ranging from Arizona to Brazil and in the Caribbean subregion from Cuba to Saint Vincent Island (from where it was described).

###### Biology.

Species of *Stantonia*, including *S. pallida*, are solitary koinobiont endoparasitoids of lepidopterans (Boughton et al. 2012), as is the case for other orgilines (Shaw and Huddleston 1991; van Achterberg 1997; Braet and Quicke 2004). Host use is known for only six of 71 species in the genus, but host range appears to be restricted to a set of moth species with similar biology.

###### Hosts.

*Stantonia pallida* has been reported from several crambids, *Diaphania hyalinata* and *D. nitidalis* (Frank and McCoy 1993), *Omiodes indicata* (Fabricius, 1775), the soybean leaffolder (Muesebeck 1938) and *Neomusotima conspurcatalis*, the Lygodium defoliator moth (Kula et al. 2010). *Stantonia pallida*, under the name *S. lamprosemae*, was imported from Colombia and released in 1991, in Florida to control melonworm and pickleworm (Frank and McCoy 1993) and was probably present before release (Kula et al. 2010). Neither *S. pallida* nor *S. lamprosemae* were mentioned in Capinera (2011).

#### Subfamily Agathidinae

##### Alabagrus texanus

Taxon classificationAnimaliaLepidopteraBraconidae

(Cresson, 1872)

F70C9FB7-E6E8-5450-8771-45FFFADDFA58

###### Notes.

This species was not collected in our survey.

###### Distribution.

The species occurs in the Nearctic and Neotropical regions, USA (from Eastern to Nebraska and south to Florida) and in Mexico.

###### Biology.

Members of the genus *Alabagrus* are solitary parasitoids of the larvae of Lepidoptera that live in concealed habitats, such as within stems or rolled leaves (Sharkey 1988). The known host range of *A. texanus* indicates that the species is polyphagous, but is exclusively associated with leaf-rolling Crambidae. Although the biology of *Alabagrus* is unknown for most species, the early instar larva is known to possess a caudal process and pseudopodia. Initially, the larva develops as an endoparasitoid and grows slowly, it then undergoes a period of rapid development and transitions to external feeding (Hummelen 1974). The larva eventually spins a cocoon; *A. texanus* typically waits for its host to spin its cocoon before producing one itself.

###### Hosts.

Only ~10 species of *Alabagrus* have known hosts. In addition to *Diaphania hyalinata*, *A. texanus* also parasitize several Crambidae species, *Microthyris anormalis* (Guenée), *Herpetogramma theseusalis* (Walker,1869), *Pantographa limata* (Grote & Robinson, 1867), *Rhectocraspeda periusalis* (Walker, 1859), *Pilocrocis ramentalis* (Lederer, 1863), *Patania silicalis* (Guenée, 1854) and *Salbia haemorrhoidalis* (Guenée, 1854).

#### Family Ichneumonidae

**Subfamily Anomaloninae**

##### Agrypon caribbaeum

Taxon classificationAnimaliaLepidopteraIchneumonidae

(Bland, 1984)

97A2A656-8C30-57F1-B923-1570C31663AA

###### Notes.

This species was not collected in our survey.

###### Distribution.

The genus *Agrypon* comprises eight species and has a worldwide distribution. *Agrypon caribbaeum* was described from the Virgin Islands, within the Caribbean subregion of the Neotropics. The species was imported into Florida in 1983 as a potential biocontrol agent against *Diaphania hyalinata* and *D. nitidalis*, but was not released (Jansson and Pena 1990).

###### Biology.

The detailed biology of *A. caribbaeum* is unknown; however, species of *Agrypon* are known as solitary koinobiont endoparasitoids of larvae of Lepidoptera, Hymenoptera and Diptera. They are larvo-nymphal parasitoids, females oviposit in mature larvae, and adult emergence occurs during the pupal stage of the host (Barron 1989; Žikić et al. 2022).

###### Hosts.

*Agrypon* species appear to have relatively broad host range. However, *Agrypon caribbaeum* was only reared from pupae of the melonworm, *D. hyalinata* (Bland 1984).

#### Subfamily Cremastinae

##### Eiphosoma dentator

Taxon classificationAnimaliaLepidopteraIchneumonidae

(Fabricius, 1804)

B6788E31-0CA0-5A0D-A7A1-F5E4A4E7E5CF

Fig. 9

###### Notes.

This species was collected in our survey. It is cited here for the first time from Guadeloupe.

**Figure 9.**
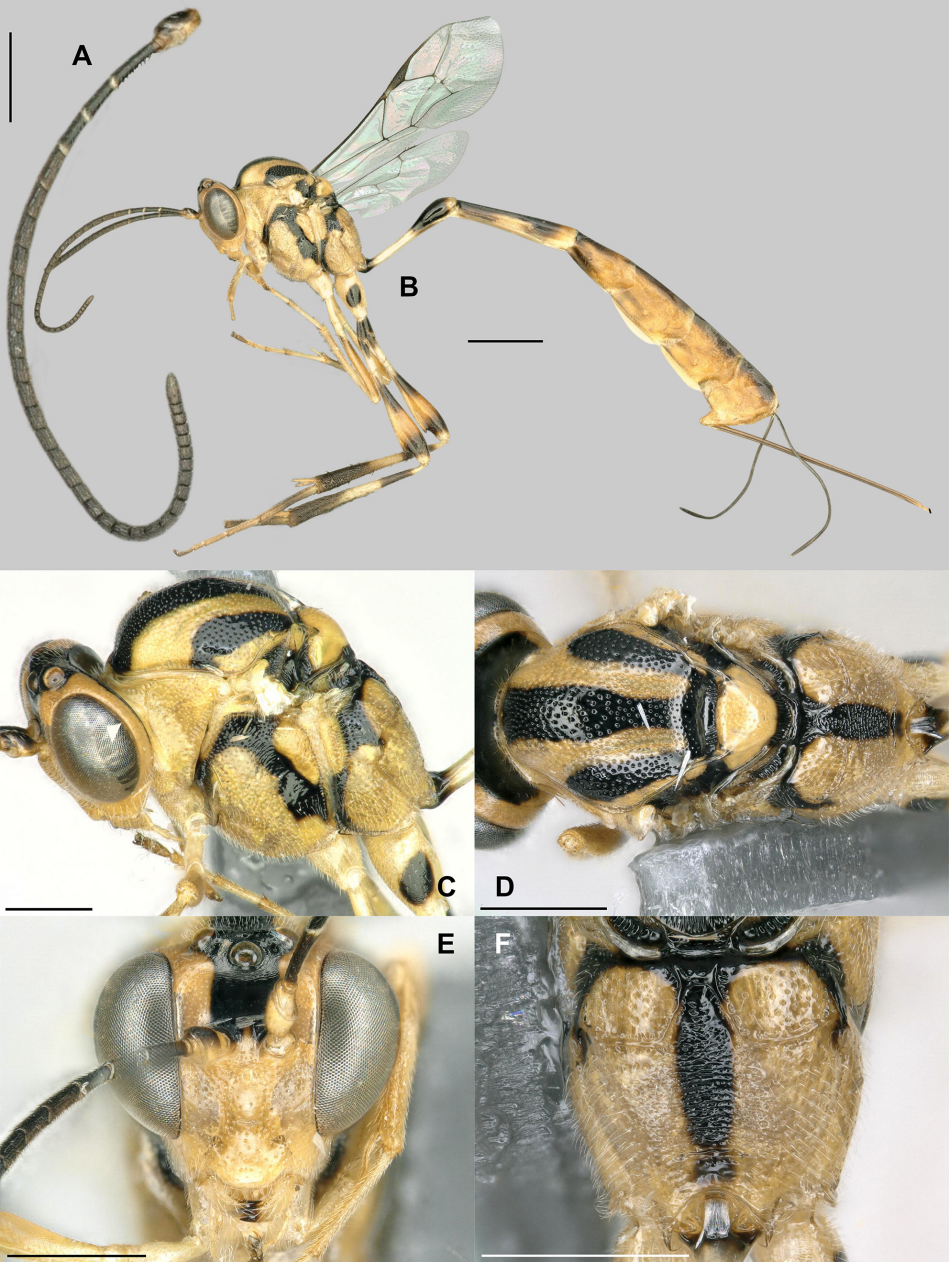
*Eiphosoma dentator*, ♀. **A**. Antenna; **B**. Habitus, lateral view; **C**. Mesosoma, lateral view; **D**. Mesosoma, dorsal view; **E**. Head, frontal view; **F**. Propodeum. Scale bars: 500 µm (**A, C–G**); 1 mm (**B**).

###### Material examined.

Guadeloupe • 1 ♀; Petit–Bourg, INRAE experimental station; 16°12.22'N, 61°39.43'W; 6 Dec 2023; M. Gumbau leg.; reared from field-collected larvae of *Diaphania hyalinata* on cucumber; (ISA37449_D2); NCBI accession PZ362384. • 8 specimens; Abymes, commercial market garden farm; 16°16.83'N, 61°30.74'W; 6 Sept 2024; M. Gumbau leg.; reared from field-collected larvae of *Diaphania hyalinata* on cucumber; specimens stored in CBGP Cryobox MG-AG24-JF. • 6 specimens; Petit-Bourg, commercial market garden farm; 16°13.82'N, 61°36.14'W; 27 Sept 2024; M. Gumbau leg.; reared from field-collected larvae of *Diaphania hyalinata* on cucumber; specimens stored in CBGP Cryobox MG-AG24-SM.

###### Distribution.

*Eiphosoma* Cresson, 1865 is a neotropical genus of Cremastinae comprising 61 valid species. *Eiphosoma dentator* occurs from western North America south to Argentina, and from Cuba to other Caribbean islands.

###### Biology.

The species is a solitary endoparasitoid of lepidopteran larvae (Dasch 1979). Like closely relative species, *E. dentator* may parasitize first and second instar larvae and kills them when they are in their last instar. *E. dentator* prefers arid habitats and its maximum activity occurs in summer, while most neotropical ichneumonids peak between fall and spring.

###### Hosts.

*Eiphosoma dentator* mostly parasitizes Crambidae, among them several pests, such as *Antigastra catalaunalis* (Duponchel, 1833); *Loxomorpha flavidissimalis* (Grote, 1877), a potential pest in prickly pear crops (Gaona-García et al. 2020); *Lineodes interga* (Zeller, 1873), the eggplant leafroller moth (Dasch 1979); *Maruca vitrata* (F., 1787), the maruca pod borer (Muniappan et al. 2012) and was first cited as a parasitoid of *D. hyalinata* by Alam (1986) in Barbados.

##### Eiphosoma kelpanum

Taxon classificationAnimaliaLepidopteraIchneumonidae

(Gauld, 2000)

9887697E-1328-531C-82F0-870E6D41BE70

###### Notes.

This species was not collected in our survey.

###### Distribution.

The species occurs in the Mesoamerican and Caribbean subregions: Costa Rica, Mexico, Trinidad & Tobago, Venezuela (González–Moreno and Bordera 2011).

###### Biology.

The species is probably a solitary endoparasitoid of lepidopteran larvae, but nothing is known about its biology apart from a single recorded host (Yu et al. 2016).

###### Hosts.

One exemplar was obtained from the larvae of *Diaphania hyalinata* in Costa Rica (Gauld et al. 2000; Yu et al. 2016).

##### Eiphosoma nigrovittatum

Taxon classificationAnimaliaLepidopteraIchneumonidae

(Cresson, 1865)

EF12162E-84E3-5833-9582-7571905E664A

###### Notes.

This species was not collected in our survey.

###### Distribution.

The species is widely distributed in the neotropics, from the USA to Brazil, including Mexico, Costa Rica, Guatemala, Venezuela, and Peru. It has also been reported from the Caribbean subregion (Cuba, Jamaica, Puerto Rico, and Trinidad & Tobago).

###### Biology.

The species is a solitary endoparasitoid of lepidopteran larvae (Dasch 1979).

###### Hosts.

*Eiphosoma nigrovittatum* is a polyphagous parasitoid mostly parasitizing Crambidae such as *Alatuncusia* sp., *Diaphania indica*, *D. hyalinata*, *Herpetogramma bipunctalis* (Fabricius, 1794), *Lineodes* sp., *Spoladea recurvalis* (Fabricius, 1787) and *Symphysa lepidaria* (Stoll in Cramer & Stoll, 1781).

##### Eiphosoma oyafusoi

Taxon classificationAnimaliaLepidopteraIchneumonidae

(Onody, Melo, Penteado-Dias & Dias-Filho, 2009)

77B881A2-76BA-54BF-823F-04BED4D68EA5

###### Notes.

This species was not collected in our survey.

###### Distribution.

Only known from Brazil.

###### Biology.

Unknown, but likely close to the biology of *Eiphosoma dentator*.

###### Hosts.

One exemplar was obtained from the larvae of *Diaphania hyalinata* Linnaeus (Lepidoptera, Crambidae) collected in *Eruca sativa* Miller organic cultivars (Onody et al. 2009).

##### Pristomerus spinator

Taxon classificationAnimaliaLepidopteraIchneumonidae

(Fabricius, 1804)

35F818F9-7A5E-53FB-B496-31DE01417DE1

###### Notes.

This species was not collected in our survey.

###### Distribution.

*Pristomerus spinator* is widely distributed in the Western Hemisphere from northern USA to Brazil, including Meso-America (Costa Rica, Honduras, Nicaragua).

###### Biology.

The species is a koinobiont endoparasitoid of Lepidoptera larvae. Female deposit a single egg in a host larva typically at third to fourth instar. Female can produce between 200 and 350 eggs (Domínguez-Jiménez et al. 2000). The development time from egg to adult ranges from 15 to 20 days. The host larva dies at the prepupal stage when the parasitoid larva emerges and spins a whitish or brownish cocoon in which it pupates. Adults are commonly associated with low vegetation (Cave 1995).

###### Hosts.

The species is highly polyphagous and attacks a wide range of Lepidoptera, including several families (Crambidae, Gelechiidae, Noctuidae, Pieridae, Tortricidae) as well as pests of agriculture (*Ostrinia nubilalis* Hübner, 1796, the European corn borer; *Helicoverpa armigera* Hübner, 1808; *Helicoverpa zea* Boddie, 1850 and *Spodoptera frugiperda* Smith, 1797). The species was cited from *D. hyalinata* by Capinera (2001) in Florida.

#### Subfamily Pimplinae

##### Itoplectis conquisitor

Taxon classificationAnimaliaLepidopteraIchneumonidae

(Say, 1836)

4D2EA37D-68AC-5ACD-848E-62D18EE548F1

###### Notes.

This species was not collected in our survey.

###### Distribution.

*Itoplectis conquisitor* is widely distributed throughout the Americas, ranging from Pennsylvania to Florida, and from British Columbia to Central America and Ecuador.

###### Biology.

Species of *Itoplectis* are solitary idiobiont endoparasitoid wasps. They are synovigenic, producing relatively large, yolk-rich eggs and typically carrying only a few mature eggs at any given time during oviposition periods (Ueno and Ueno 2007). As a result, *Itoplectis* species, and Pimplinae in general, are host-feeders (Ueno 1999). Development from egg to adult requires ~20 days (Townes and Townes 1960). The reported parasitism rate of this species rarely exceeded 1% (Hudon 1966).

###### Hosts.

As with most idiobiont parasitoids, species of *Itoplectis* are polyphagous and parasitize a broad range of lepidopteran pupae and prepupae (Ueno and Ueno 2007). *Itoplectis* species are also parasitoids of Hymenoptera (Tenthredinoidea and Ichneumonoidea), rarely in puparia of Diptera and in pupae of Coleoptera. *Itoplectis conquisitor* is known to attack nearly any exposed or weakly concealed lepidopteran pupa or prepupa found on trees, shrubs, or herbaceous plants. It has been recorded parasitizing species from numerous Lepidoptera families, including *D. hyalinata* (Townes and Townes 1960). This species can also act as a facultative hyperparasitoid, developing at the expense of a primary parasitoid (Ichneumonidae or Braconidae) and can also serve as host itself. This species attacks almost any exposed or weakly protected lepidopterous pupa or prepupa on trees, shrubs, or herbage.

##### Neotheronia bicincta

Taxon classificationAnimaliaLepidopteraIchneumonidae

(Cresson, 1865)

29F12025-0BD4-5C1D-BAA5-B3DF1B66D20C

###### Notes.

This species was not collected in our survey.

###### Distribution.

The neotropical genus *Neotheronia* Krieger, 1899 includes 72 valid species (Yu et al. 2016). *Neotheronia bicincta* occurs in the southeastern USA, Cuba, and Costa Rica.

###### Biology.

Species of the *Neotheronia* are primary idiobiont ectoparasitoids of Lepidoptera pupae and prepupae within cocoon. They can also be facultative hyperparasitoids via primary parasitoids belonging to Ichneumonidae or Tachinidae (Diptera) (Gauld 1991; Gauld et al. 1998). According to Gauld (1991), *Neotheronia* species are known to parasitize Pimplinae species.

###### Hosts.

The species was obtained from larvae of *D. hyalinata* in Cuba (Myers 1932).

##### Pimpla golbachi

Taxon classificationAnimaliaLepidopteraIchneumonidae

(Porter, 1970)

EEA1D6C5-0C28-5D4F-B7FB-CA2416F299B1

###### Notes.

This species was not collected in our survey.

###### Distribution.

*Pimpla golbachi* is widely distributed across South America including Argentina, Bolivia, Brazil, Colombia, Paraguay, and Uruguay (Villanueva-Bonilla et al. 2021).

###### Biology.

Species of the diverse genus *Pimpla* (~200 species) are idiobiont endoparasitoids, mainly of lepidopteran prepupae or pupae (Ueno 1999; Quicke 2014). *Pimpla* species are synovigenic, and females that exhibit host-feeding behavior deposit only a few mature eggs in the host larva (Ueno and Ueno 2007). Development from egg to adult takes 20 to 25 days and includes five larval instars (Rojas-Rousse and Benoit 1977).

###### Hosts.

As typical for idiobiont parasitoids, *Pimpla* species are polyphagous and parasitize a wide range of lepidopteran pupae and prepupae (Ueno and Ueno 2007). *Pimpla golbachi* has been recorded parasitizing several moth families, including *Diaphania hyalinata* (De Santis 1980); *Pectinophora gossypiella* (Saunders, 1844), the pink bollworm (Gelechiidae); *Alabama argillacea* (Hübner, 1818), the cotton leafworm (Noctuidae) (Porter 1970); *Colias lesbia* (Fabricius, 1775), the Lesbia clouded yellow (Pieridae) (Avalos et al. 2011); *Rhyacionia buoliana* (Denis & Schiffermüller, 1775), the pine shoot moth (Porter 1970) (Tortricidae).

##### Pimpla marginella

Taxon classificationAnimaliaLepidopteraIchneumonidae

(Brullé, 1846)

BBE803C3-F76E-5F99-AC0F-E305BCC287A6

###### Notes.

This species was not collected in our survey.

###### Distribution.

The species occurs in the Nearctic and Caribbean subregion, from coastal Georgia through Florida (USA) reaching Jamaica and Puerto Rico in the Caribbean Islands.

###### Biology.

Like other species of *Pimpla*, this species is a solitary idiobiont endoparasitoid of lepidopteran larvae (Myers 1932).

###### Hosts.

*Pimpla marginella* has been reported to parasitize pupae of *Neomusotima conspurcatalis* Warren (Lepidoptera: Crambidae), a biological control agent of *Lygodium microphyllum* (Cav.) R. Br. (Polypodiales: Lygodiaceae) in Florida, but also from *D. hyalinata* (Myers 1931, 1932).

#### Subfamily Campopleginae

##### Casinaria infesta

Taxon classificationAnimaliaLepidopteraIchneumonidae

(Cresson, 1872)

68DAF9B4-9644-52F0-A250-3625DE96AAA8

###### Notes.

This species was not collected in our survey.

###### Distribution.

*Casinaria* Holmgren, 1859 is a relatively diverse genus of Campopleginae, comprising ~135 species. It has a cosmopolitan distribution, but occurs mostly across the Holarctic region. *Casinaria infesta* is recorded in the USA (Alabama, Kansas, Texas, Florida to Maryland) and has also been introduced to Hawaii (Reimer and Beardsley 1986).

###### Biology.

*Casinaria infesta* is a koinobiont, a solitary endoparasitoid associated with the larval stages of a restricted set of Lepidoptera. This biology may confer a relatively restricted range of hosts to *Casinaria* species.

###### Hosts.

In addition to *Diaphania hyalinata*, reported as a host in Florida (Pena et al. 1987), the species also parasitizes various Crambidae species including the polyphagous *Udea rubigalis* (Guenée, 1854), commonly known as the celery leaftier; *Loxomorpha flavidissimalis* (Grote, 1878), the “Opuntia webworm” used in biological control against *Opuntia* cacti (Kaufman and Wright 2011), and *Spoladea recurvalis* (Fabricius, 1775), the beet webworm, a widespread pest of spinach and other crops (Kaufman and Wright 2011).

##### Diadegma pattoni

Taxon classificationAnimaliaLepidopteraIchneumonidae

(Ashmead, 1890)

A64E6F49-FA9A-568B-B43B-75519B0E4E4E

###### Notes.

This species was not collected in our survey.

###### Distribution.

The species occurs from the Nearctic region to the Mesoamerican subregion, from the Great Lakes to Florida, Costa Rica, and Panama. It was introduced in Hawaii either accidentally or purposely for control of lepidopterous pests (Beardsley 1977).

###### Biology.

Like many other species of *Diadegma* associated with lepidopteran larvae, this species may probably be a koinobiont solitary endoparasitoid (Pourian et al. 2015).

###### Hosts.

The species is known to be a generalist parasitoid, parasitizing the corn earworm *Helicoverpa zea* (Boddie, 1850) (Noctuidae) (Maes 1999), the garden webworm (*Achyra rantalis* (Guenée, 1874) (Crambidae) (Capinera 2001). The species was obtained from the larvae of *Diaphania hyalinata* Linnaeus (Lepidoptera, Crambidae) (Yu et al. 2016).

#### Subfamily Cryptinae

##### Gambrus ultimus

Taxon classificationAnimaliaLepidopteraIchneumonidae

(Cresson, 1864)

1E55E56A-56F2-5C05-9512-9A2A67CD778E

###### Notes.

This species was not collected in our survey.

###### Distribution.

*Gambrus* Förster, 1868 is a small Holarctic genus comprising 36 species, with nine described from the American continent. *Gambrus ultimus* is distributed across the northeastern USA (e.g., New Hampshire, Pennsylvania) but its range extends westward to Colorado and southward to Mexico in the Mesoamerican subregion.

###### Biology.

The biology of *Gambrus* species remains poorly known. They appear to be koinobiont endoparasitoids of various moths. Rearing indicates that *G. ultimus* parasitizes the prepupal or pupal stages of its hosts. The mature larva of *G. ultimus* exits the host pupa and spins an elongate silken cocoon adjacent to the shriveled remains of the host (Brushwein and Childers 1990).

###### Hosts.

Known hosts encompass species of Crambidae such as *D. hyalinata* cited in Capinera (2001), as well as Noctuidae and Erebidae. Cassani (1985) reared *G. ultimus* from pupae of *Acronicta insularis* (Herrich-Schäffer, 1868) (Noctuidae). Similarly, Hall (1985) also recovered *G. ultimus* from pupae of field-collected grassloopers, *Mocis latipes* Guenée, 1852 (Erebidae) and Brushwein and Childers (1990) obtained the wasp from *Selenisa sueroides* Guenée, 1852 (Erebidae). The species have also been cited as a parasitoid of *Hypera* sp. (Curculionidae) (Puttler et al. 1973).

##### Polycyrtus giacomellii

Taxon classificationAnimaliaLepidopteraIchneumonidae

(Schrottky, 1911)

72FF4C03-3D0A-52C8-ABC0-782D7E3F23EE

###### Notes.

This species was not collected in our survey.

###### Distribution.

*Polycyrtus* Spinola, 1840 is a diverse genus of Ichneumonidae, with ~170 species occurring in the Neotropics (Mexico, Peru, Panama, Brazil) (Cushman 1931; Carrasco 1972; Kasparyan and Ruiz-Cancino 2003).

###### Biology.

Very little is known on the biology of this species, but it could be a koinobiont endoparasitoids of various moths.

###### Hosts.

*Polycyrtus giacomellii* has been reared from *D. hyalinata* in Brazil (De Santis 1980).

##### Polycyrtus lituratus

Taxon classificationAnimaliaLepidopteraIchneumonidae

(Brullé, 1846)

6B4A1E39-9EE0-55AC-980E-F937493AD8A0

###### Notes.

This species was not collected in our survey.

###### Distribution.

The species is only known from Cuba and Hispaniola (Cushman 1931; Perez-Gelabert 2008).

###### Biology.

Very little is known on the biology of this species, but it could be a koinobiont endoparasitoids of various moths.

###### Hosts.

*Polycyrtus semialbus* has been reared from *D. hyalinata* in Cuba (King and Saunders 1984).

##### Polycyrtus semialbus

Taxon classificationAnimaliaLepidopteraIchneumonidae

(Cresson, 1865)

C97A6A8F-B9F0-54CC-8458-26EFA4F7FB25

###### Notes.

This species was not collected in our survey.

###### Distribution.

The species is only known from Mexico and Cuba (Kasparyan and Ruiz-Cancino 2003).

###### Biology.

Very little is known on the biology of this species, but it could be a koinobiont endoparasitoids of various moths.

###### Hosts.

*Polycyrtus semialbus* has been reared from *D. hyalinata* in Cuba (King and Saunders 1984).

###### Comment.

An undetermined species of *Polycyrtus* from Colombia has been imported in 1984 in Florida as a potential biocontrol agent for *D. hyalinata* and *D. nitidalis*, but it was never released (Jansson and Pena 1990).

For completeness, we note a citation of two ichneumonid species associated with *Diaphania hyalinata* in Argentina (De Santis et al. 2008), which refers to a technical note by Pelicano et al. (1989) that we were unable to find. The species mentioned are *Casinaria bonaerensis* (Schrottky, 1902) and *Parania triannulata* (Fabricius) (Anomaloninae). Similarly, the mention of *Spilochalcis diaphaniae* (Ashmead) in Jansson and Pena (1990) is problematic as this name does not appear to exist. *Conura acragae* (Chalcididae) is listed as a parasitoid of *Diaphania hyalinata* in Cave (1995), but not in UCD Community (2023). It appears together with *Brachymeria ovata* (Say), *Cotesia marginiventris* (Cresson), *ltoplectis conquisitor* (Say) and *Catolaccus aeneoviridis* (Girault). However, several of these identifications are questionable, and we have therefore chosen not to consider these records.

### *Diaphania hyalinata* - parasitoid interactions in Guadeloupe

Of the 37 parasitoids of *D. hyalinata* identified in our review of the literature for the neotropics, eight species were recovered during our field survey in Guadeloupe, all of which are new records for the Island (Fig. 1, Table 1). Four likely emerged from larvae/pupae of the moth: *Apanteles impiger*, *Eiphosoma dentator*, *Schoenlandella montserratensis*, and *Trichospilus diatraeae*. *Aphanogmus fijiensis* was likely a hyperparasitoid of *Apanteles impiger*, and two could either parasitize the moth or act as hyperparasitoids (*Brachymeria ovata* that is highly polyphagous and maybe *Conura ferruginea* that acts as a facultative hyperparasitoid in some cases). *Trichogramma pretiosa* was reared from eggs of *D. hyalinata*. It is difficult to determine whether the other parasitoids identified in our review have not arrived in Guadeloupe, or if the scope of our study (location, number of specimens of *Diaphania* collected, and survey duration) simply did not allow us to sample them. That being said, Guadeloupe is a small and relatively isolated island, and considering this context, identifying eight species of a possible 37 is already quite significant.

**Table 1.** Results of our field survey in Guadeloupe. Number of *D. hyalinata* specimens collected in the fields, status and fate of specimens and parasitism assessment. * Gregarious species (total count as occurrence for *A. fijiensis* and *T. diatreae* and count in number for *T. pretiosa*). † hyperparasitoid species.

**Variable**	**Description**	**Total**	**Outcome ( %)**	**Relative abundance ( %)**
*D. hyalinata* larvae and pupae	Larvae and pupae of *D. hyalinata* collected in the field	953	100	
Outcome: moth	Moths emerged from *D. hyalinata* larvae and pupae	154	16.16	
Outcome: parasitoid	Parasitoid wasps emerged from *D. hyalinata* larvae and pupae	111	11.65	
*Schoenlandella montserratensis*	Ichneumonoidea: Braconidae	76	7.97	68.47
*Eiphosoma dentator*	Ichneumonoidea: Ichneumonidae	15	1.57	13.51
*Conura ferruginea*	Chalcidoidea: Chalcididae	7	0.73	6.31
*Brachymeria ovata*	Chalcidoidea: Chalcididae	3	0.31	2.70
*Apanteles impiger*	Ichneumonoidea: Braconidae	2	0.21	1.80
Trichospilus diatraeae *	Chalcidoidea: Eulophidae	1	0.10	0.90
Aphanogmus fijiensis * †	Ceraphronoidea: Ceraphronidae	2	0.21	1.80
*D. hyalinata* eggs	Eggs of *D. hyalinata* collected in the fields	248	100	
Outcome: larvae	Larvae emerged from *D. hyalinata* eggs	0	0	
Outcome: Trichogramma pretiosa *	Chalcidoidea: Trichogrammatidae	113	45.56	100

Unfortunately, we faced a low level of outcomes in our rearing (378 outcomes for 1,201 specimens collected). Nevertheless, of the 953 larvae and pupae that could be maintained, 154 produced a moth, while 111 yielded one or several parasitoid(s). More than half of the parasitoids that emerged from the larvae and pupae were identified as *Schoenlandella montserratensis*, which represents a parasitism rate of 8% relative to the total number of sampled *D. hyalinata* specimens. The second most abundant parasitoid was *Eiphosoma dentator*, accounting for nearly 14% of the parasitoids that emerged.

Although we were unable to determine the initial parasitism rate of *D. hyalinata* eggs collected in the field, *Trichogramma pretiosa* appears capable of achieving notably high levels of parasitism (46%). With *Schoenlandella montserratensis*, provided it can be reared efficiently, these two species could represent promising candidates for the biological control of *D. hyalinata* in Guadeloupe. That said, due to the gregarious behavior of *Trichogramma pretiosa*, the ratio of parasitoids per egg could not be correctly assessed, and the rate of parasitism could be lower. Finally, unparasitized eggs are transparent, which may have limited their collection in the field, creating a bias in the evaluation of egg parasitism.

It may also be worthwhile to precisely evaluate the effectiveness of *Eiphosoma dentator* and *Apanteles impiger* in controlling the moth, as well as the feasibility of rearing them, while assessing their potential non-target effects on local fauna. However, the presence of the hyperparasitoid *Aphanogmus fijiensis* should be taken into account, as it may compromise biocontrol efforts by parasitizing the released parasitoids (Cochereau 1991). Given that the two Chalcididae tend to be more generalist and may act as hyperparasitoids, they appear to be less suitable candidates for the biocontrol of *D. hyalinata*, particularly due to the risk of unintended impacts on native fauna. Overall, it would be valuable to improve our understanding of the biological and environmental factors, such as agricultural practices and landscape features, which enhance the natural regulatory role of this parasitoid community.

Finally, among the eight species studied, only six were barcoded (COI could not be amplified for two species: *Brachymeria ovata* and *Trichospilus diatraeae*, possibly because the specimens were not well preserved). However, only one (*Trichogramma pretiosa*) had reference sequences available on NCBI (percentage identity = 99.6%, accession KF573380, uploaded by taxonomists from the UC Riverside CA USA). Sequences labelled as *Eiphosoma dentator* from Mexico were also retrieved (accessions: MZ196444 and MZ196445). However, the pairwise identity with our sequences was only 95.1%, suggesting that this species could be a complex.

This once again highlights the limited completeness of reference databases for a group as ecologically significant as parasitoids, which play a key role in regulating many agricultural pest species worldwide. Therefore, maintaining taxonomic expertise on these groups remains essential for the success of biological control programs.

## Supplementary Material

XML Treatment for Aphanogmus fijiensis

XML Treatment for Brachymeria ovata

XML Treatment for Brachymeria mnestor

XML Treatment for Brachymeria flavipes

XML Treatment for Conura ferruginea

XML Treatment for Conura immaculata

XML Treatment for Conura hirtifemora

XML Treatment for Conura side

XML Treatment for Palmistichus elaeisis

XML Treatment for Trichospilus diatraeae

XML Treatment for Trichogramma atopovirilia

XML Treatment for Trichogramma pretiosa

XML Treatment for Trichogramma exiguum

XML Treatment for Apanteles impiger

XML Treatment for Hypomicrogaster areolaris

XML Treatment for Chelonus busckiella

XML Treatment for Chelonus junkoshimurae

XML Treatment for Schoenlandella diaphaniae

XML Treatment for Schoenlandella montserratensis

XML Treatment for Stantonia pallida

XML Treatment for Alabagrus texanus

XML Treatment for Agrypon caribbaeum

XML Treatment for Eiphosoma dentator

XML Treatment for Eiphosoma kelpanum

XML Treatment for Eiphosoma nigrovittatum

XML Treatment for Eiphosoma oyafusoi

XML Treatment for Pristomerus spinator

XML Treatment for Itoplectis conquisitor

XML Treatment for Neotheronia bicincta

XML Treatment for Pimpla golbachi

XML Treatment for Pimpla marginella

XML Treatment for Casinaria infesta

XML Treatment for Diadegma pattoni

XML Treatment for Gambrus ultimus

XML Treatment for Polycyrtus giacomellii

XML Treatment for Polycyrtus lituratus

XML Treatment for Polycyrtus semialbus

## Additional information

### Artificial Intelligence (AI) use

The authors accept full responsibility for the content of the manuscript, including the disclosure of any use of AI.

No AI tools were used in the preparation of this manuscript.

### Author ORCIDs

M. Gumbau https://orcid.org/0000-0002-6570-3386

C. Marteil https://orcid.org/0009-0008-0478-1082

G. Groussier https://orcid.org/0009-0004-6981-7227

A. Cruaud https://orcid.org/0000-0001-8932-4199

S. Nidelet https://orcid.org/0000-0003-0437-319X

S. Warot https://orcid.org/0009-0006-0678-9008

J. Fernandez-Triana https://orcid.org/0000-0003-0425-0309

L. Penet https://orcid.org/0000-0003-1512-6303

J.-M. Blazy https://orcid.org/0000-0002-4132-0217

J.-Y. Rasplus https://orcid.org/0000-0001-8614-6665

### Data availability

Data availability statement: Barcodes generated in this study were uploaded to NCBI (Accessions PZ362382–PZ362393).
